# Supplementary material for: Hox/Meis-dependent gene-regulatory transition underlies cardiopharyngeal neural crest diversification
Source: EMBO J. 2026 Sep 15;45(19):6978–7020. doi: 10.1038/s44318-026-00886-x (PMC13631262; doi:10.1038/s44318-026-00886-x)
Supplement: Supplementary file 1 [file 44318_2026_886_MOESM16_ESM.pdf]

Expanded View Figures

Figure EV1. Transcriptomic and chromatin accessibility profiles of diverse cell types in the cardiopharyngeal region.

(**A**) FACS gating of single-cell suspension from *Wnt1-Cre; R26R-EYFP* embryos. All events were hierarchically gated by forward scatter (FSC) and side scatter (SSC), including area (A), height (H), and width (W). FSC singlets were gated, followed by exclusion of 7-AAD-positive dead cells to sort live EYFP-positive NCCs (P3) and EYFP-negative non-NCCs (P2). (**B**) Heatmap showing differentially expressed genes (DEGs) across cardiopharyngeal cell clusters (as in Fig. 1D). DEGs with an average log2 expression >1 were ranked by adjusted *P* value from the Wilcoxon rank-sum test with Bonferroni correction, and the top ten genes per cluster were selected. Values represent cluster-averaged expression. (**C**, **D**) Heatmaps showing representative TFs differentially expressed in each cluster (**C**) and the enrichment of their binding motifs in open chromatin regions (**D**) across cardiopharyngeal cell clusters. (**E**, **F**) Heatmaps comparing differentially expressed TF genes (**E**) and the enrichment of their binding motifs in open chromatin regions (**F**) between NCC-derived and non-NCC-derived mesenchymal cells at E14.5 and E17.5.

 © The Author(s)

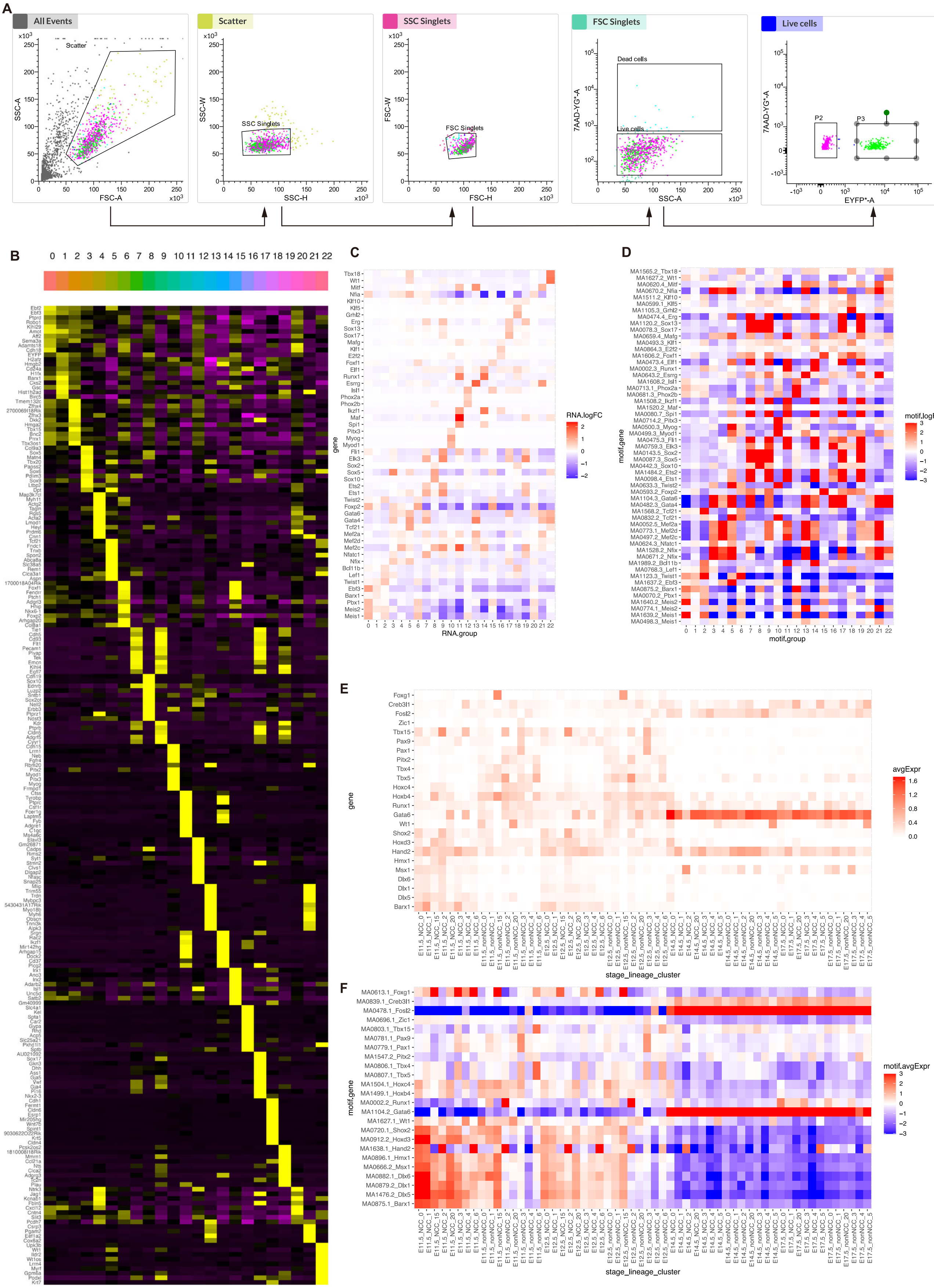
A
All Events
Scatter
SSC Singlets
FSC Singlets
Live cells
SSC-A
FSC-A
SSC-W
SSC-H
FSC-W
FSC-H
7AAD-YG*-A
EYFP*-A
Dead cells
Live cells
P2
P3
B
0 1 2 3 4 5 6 7 8 9 10 11 12 13 14 15 16 17 18 19 20 21 22
C
RNA.logFC
RNA.group
gene
D
motif.logFC
motif.group
motif.gene
E
avgExpr
gene
stage_lineage_cluster
F
motif.avgExpr
motif.gene
stage_lineage_cluster

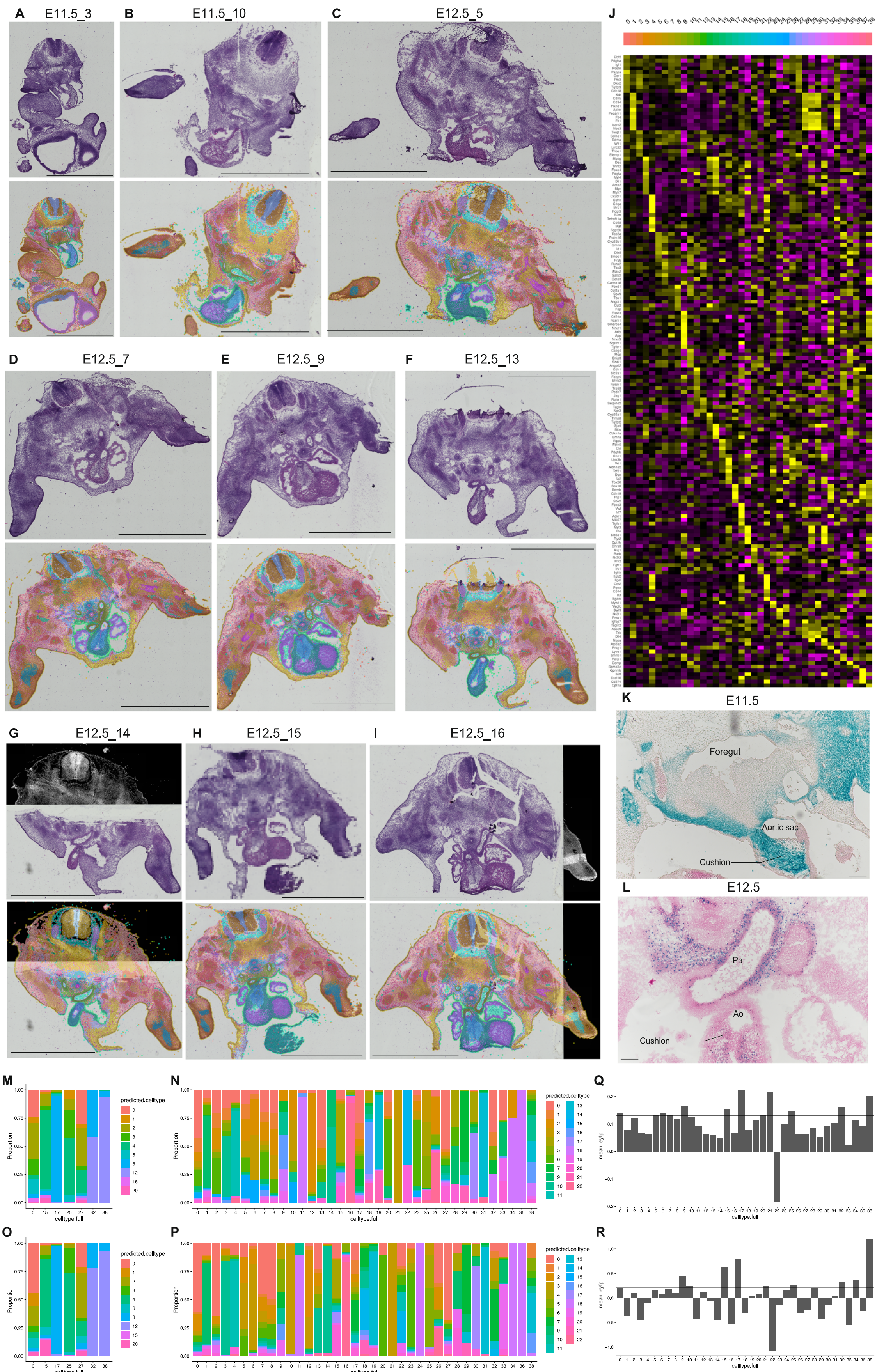
A
E11.5_3
B
E11.5_10
C
E12.5_5
D
E12.5_7
E
E12.5_9
F
E12.5_13
G
E12.5_14
H
E12.5_15
I
E12.5_16
J
K
E11.5
Foregut
Aortic sac
Cushion
L
E12.5
Pa
Ao
Cushion
M
N
O
P
Q
R
predicted.celltype
Proportion
celltype.full
mean_evlp

Figure EV2. Xenium analysis of cardiopharyngeal tissues reveals distinct transcriptomic signatures across diverse cell types.

(**A–I**) Xenium spatial transcriptomics of cardiopharyngeal tissues at E11.5 (**A**, **B**) and E12.5 (**C–I**) with hematoxylin–eosin staining. (**J**) Heatmaps showing DEGs across Xenium-identified cell clusters (as in Fig. 2E). (**K**, **L**) β-Galactosidase staining of cardiopharyngeal tissues from *Wnt1-Cre;R26R-LacZ* embryos at E11.5 (**K**) and E12.5 (**L**). (**M–P**) Comparison of NCC-derived (**M**, **N**) and non-NCC-derived (**O**, **P**) mesenchymal cell distributions between fine cluster identities (as in Fig. 1D) and RCTD-predicted cell clusters at E11.5 (**M**, **N**) and E12.5 (**O**, **P**). (**Q**, **R**) Estimated mean *EYFP* expression by Tangram across Xenium integrated clusters. The horizontal lines indicate the threshold corresponding to the 65th percentile across all spots.

© The Author(s)

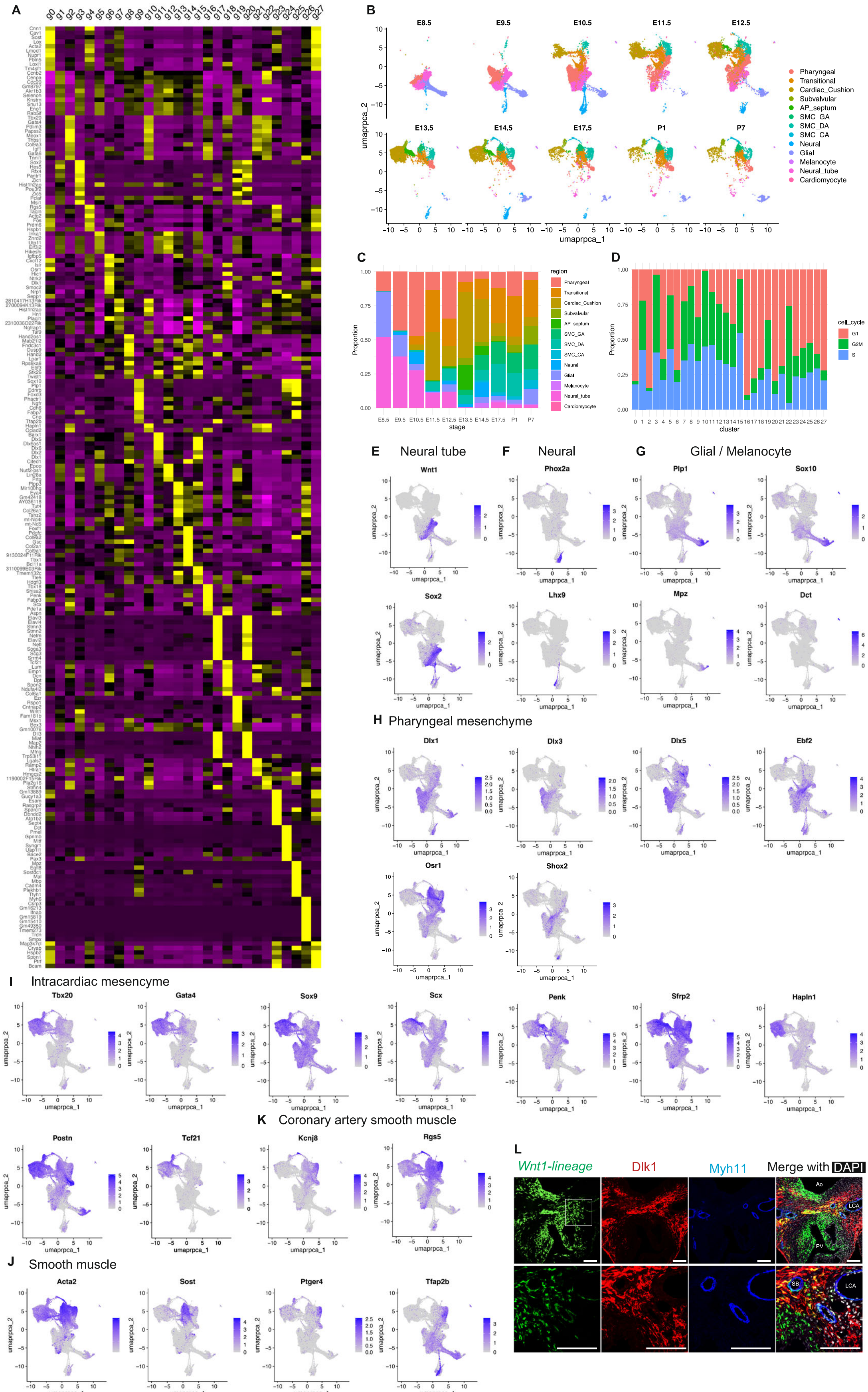
A
B
C
D
E Neural tube
F Neural
G Glial / Melanocyte
H Pharyngeal mesenchyme
I Intracardiac mesenchyme
J Smooth muscle
K Coronary artery smooth muscle
L
Wnt1-lineage
Dlk1
Myh11
Merge with DAPI
Pharyngeal
Transitional
Cardiac_Cushion
Subvalvular
AP_septum
SMC_GA
SMC_DA
SMC_CA
Neural
Glial
Melanocyte
Neural_tube
Cardiomyocyte

Figure EV3. Transcriptional and developmental profiling of NCC-derived lineages.

(**A**) Heatmap showing DEGs across integrated NCC clusters (as in Fig. 3B). DEGs with an average log2 expression > 1 were ranked by adjusted *P* value from the Wilcoxon rank-sum test with Bonferroni correction, and the top ten genes per cluster were selected. Values represent cluster-averaged expression. (**B**) UMAP decomposition of NCC subcategories (as in Fig. 4A) across sequential developmental stages. (**C**) Cell type classification at each developmental stage. (**D**) Cell cycle phase classification of each cluster. (**E–K**) Feature plots showing representative marker gene expression for distinct NCC populations: neural tube (premigratory) (**E**), neural (**F**), glial/melanocyte (**G**), pharyngeal mesenchyme (**H**), intracardiac mesenchyme (**I**), smooth muscle (great arteries and ductus arteriosus) (**J**), and coronary artery smooth muscle (**K**). (**L**) Immunostaining for Myh11 (red) and Dlk1 (blue). NCCs are marked by EYFP (green) and in *Wnt1-Cre;R26R* embryos at E17.5. Nuclei are stained by DAPI (white). Boxed regions (top) are shown at higher magnification (bottom). Scale bars, 50 µm.

© The Author(s)

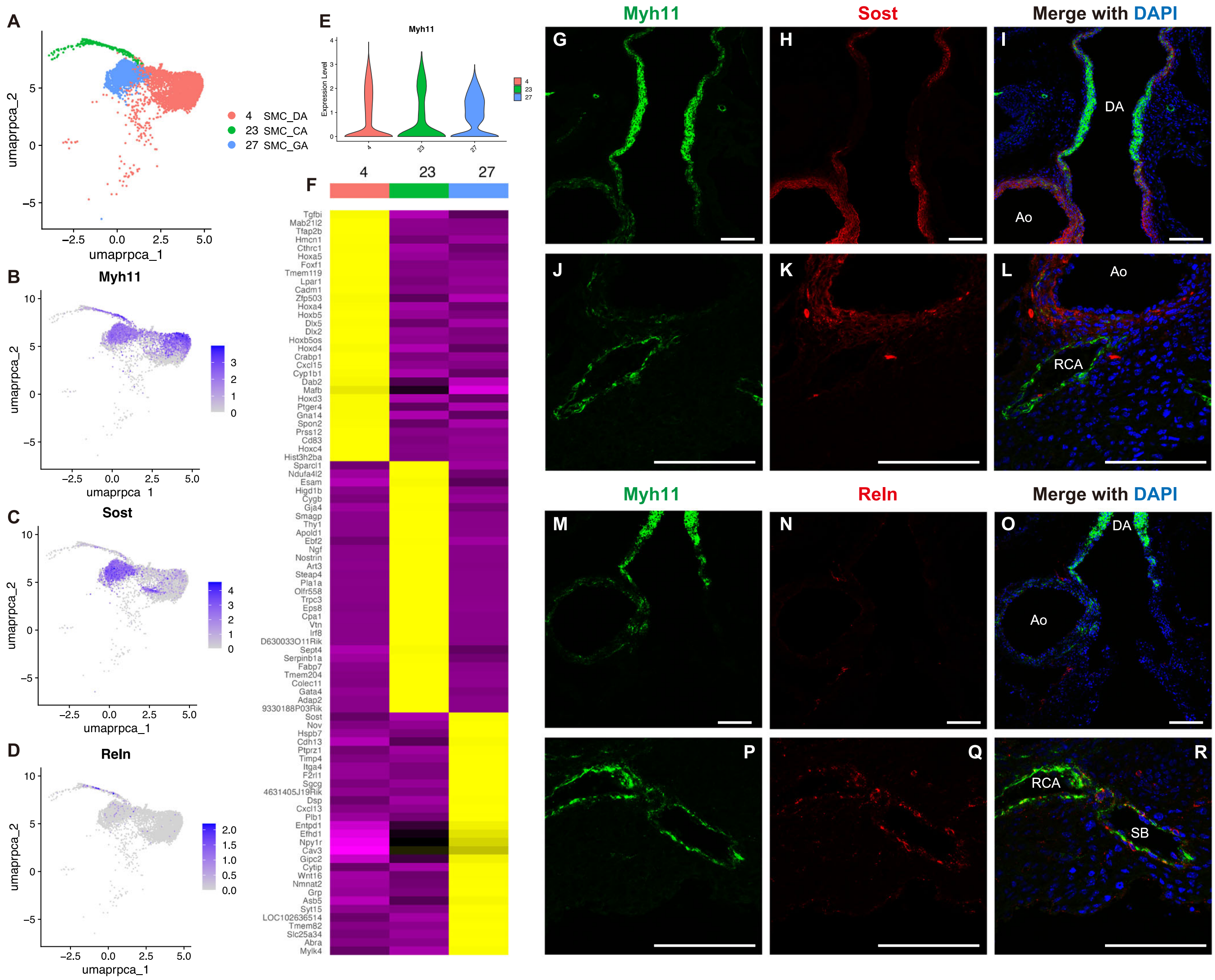

Figure EV4. Characterization of NCC-derived smooth muscle cell (SMC) subpopulations.

(**A**) UMAP plot colored corresponding SMC subpopulation. (**B–D**) Feature plots showing representative marker gene expression for distinct SMC populations: *Myh11* (mature SMCs) (**B**), *Sost* (great artery SMCs) (**C**), and *Reln* (coronary artery SMCs) (**D**). (**E**) Violin plot of *Myh11* across SMC subclusters. (**F**) Heatmap showing DEGs across SMC subpopulations. DEGs with an average log2 expression >2.5 were ranked by adjusted *P* value from the Wilcoxon rank-sum test with Bonferroni correction, and the top 30 genes per cluster were selected. Values represent cluster-averaged expression. (**G–R**) Immunostaining for Myh11 (green) (**G**, **J**, **M**, **P**) with Sost (red) (**H**, **I**, **K**, **L**) or Reln (red) (**N**, **O**, **Q**, **R**). Nuclei are stained by DAPI (blue) in E17.5 embryos. Ao aorta, DA dorsal aorta, RCA. right coronary artery; SB, septal branch. Scale bars, 50μm.

 © The Author(s)

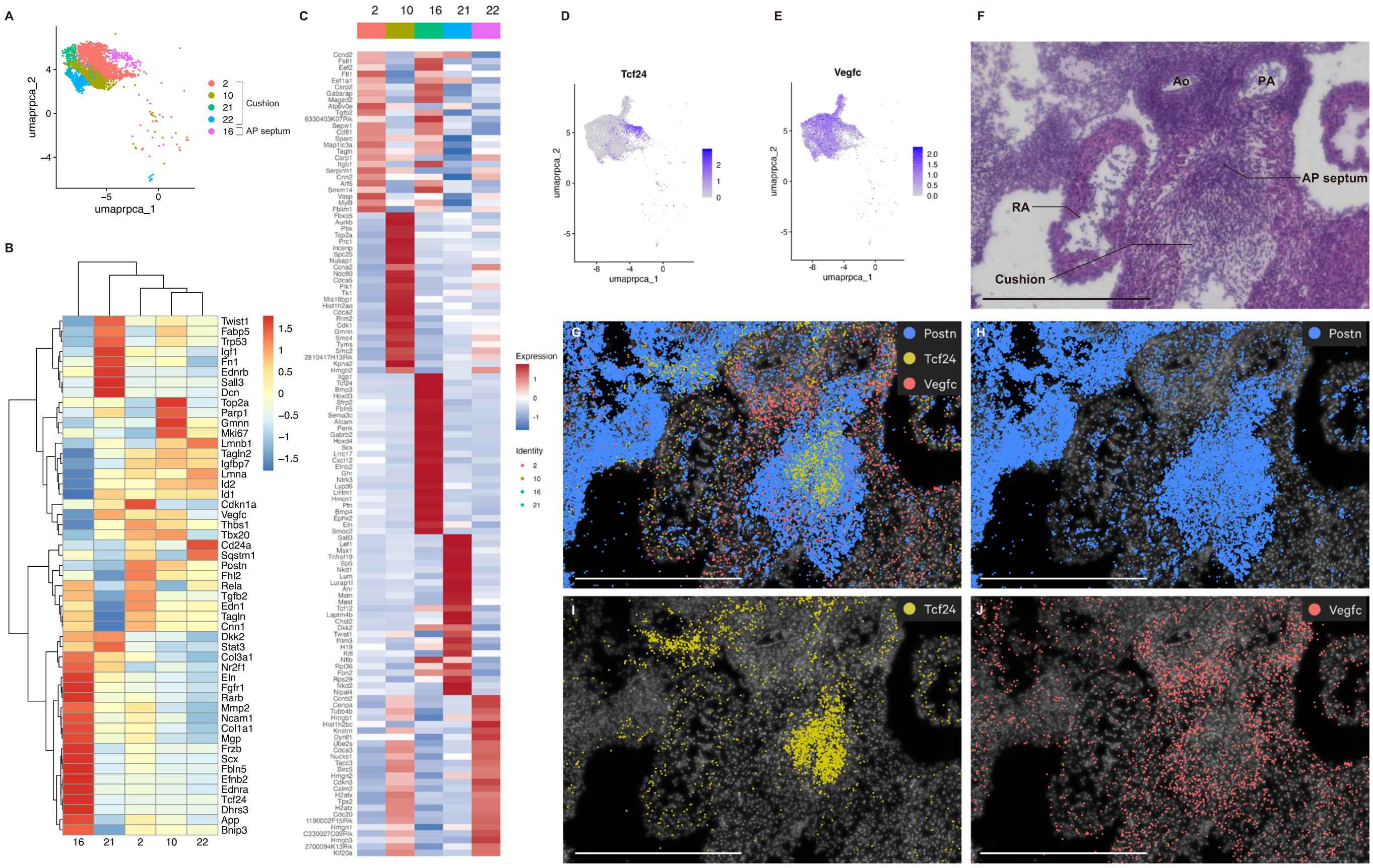

Figure EV5. Characterization of NCC-derived intracardiac mesenchyme.

(**A**) UMAP plot colored according to the intracardiac mesenchymal subpopulation. (**B**, **C**) Heatmaps showing DEGs across intracardiac mesenchymal subpopulations for genes included (**B**) or not included (**C**) in the customized Xenium panel. DEGs with an average log2 expression >0.15 were ranked by adjusted *P* value from the Wilcoxon rank-sum test with Bonferroni correction, and the top 25 genes per cluster were selected. Values represent cluster-averaged expression. (**D**, **E**) Feature plots showing the expression of *Tcf24* (**D**) and *Vegfc* (**E**). (**F–J**) Xenium spatial transcriptomic visualization of *Tcf24*, *Vegfc*, and *Postn* (**G–J**) with corresponding hematoxylin-eosin staining (**F**) in E12.5 cardiac tissue. Ao aorta, AP septum aorticopulmonary septum, PA pulmonary artery, RA right atrium. Scale bars, 500 µm.

© The Author(s)

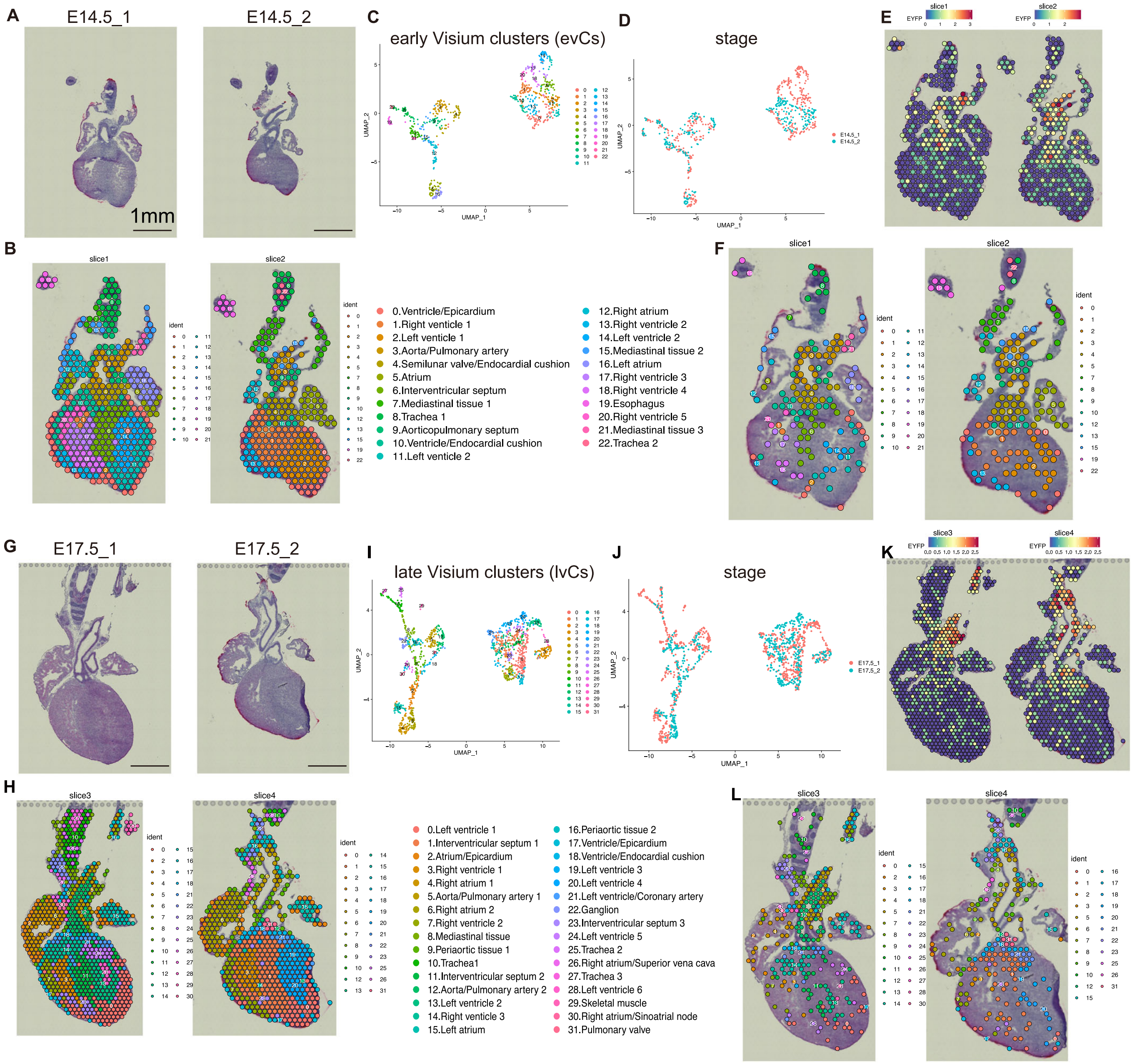

Figure EV6. Visium spatial transcriptomics of the *Wnt1-Cre*-labeled developing heart.

Spatial transcriptomic profiling using the 10x Visium platform at E14.5 (**A–F**) and E17.5 (**G–L**). On hematoxylin-eosin-stained images (**A**, **G**), clustering profiles are visualized (**B**, **H**) with UMAP plots, colored corresponding to early Visium clusters (evCs) (**C**), late Visium clusters (lvCs) (**I**), and stages (**D**, **J**). EYFP-expressing spots are overlaid on the tissue sections (**E**, **K**), with corresponding cluster annotations (**F**, **L**).

 © The Author(s)

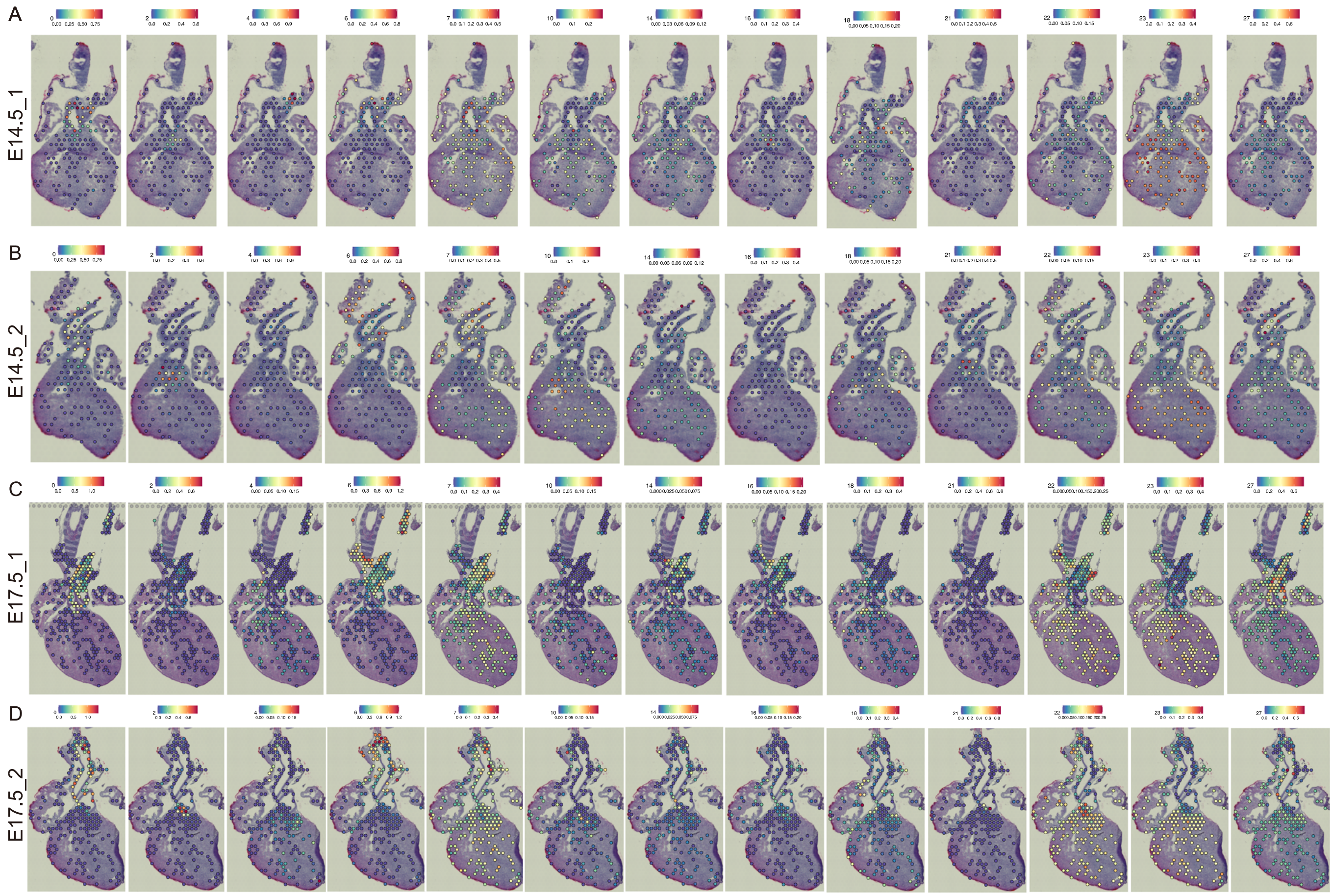

Figure EV7. Spatial localization of NCC-derived mesenchymal clusters on the Visium datasets.

Spatial mapping of NCC-derived mesenchymal clusters on the Visium datasets at E14.5 (**A**, **B**) and E17.5 (**C**, **D**), highlighting the predicted correspondence between clusters and spatial spots. Color bars represent the probability of spatial prediction.

© The Author(s)

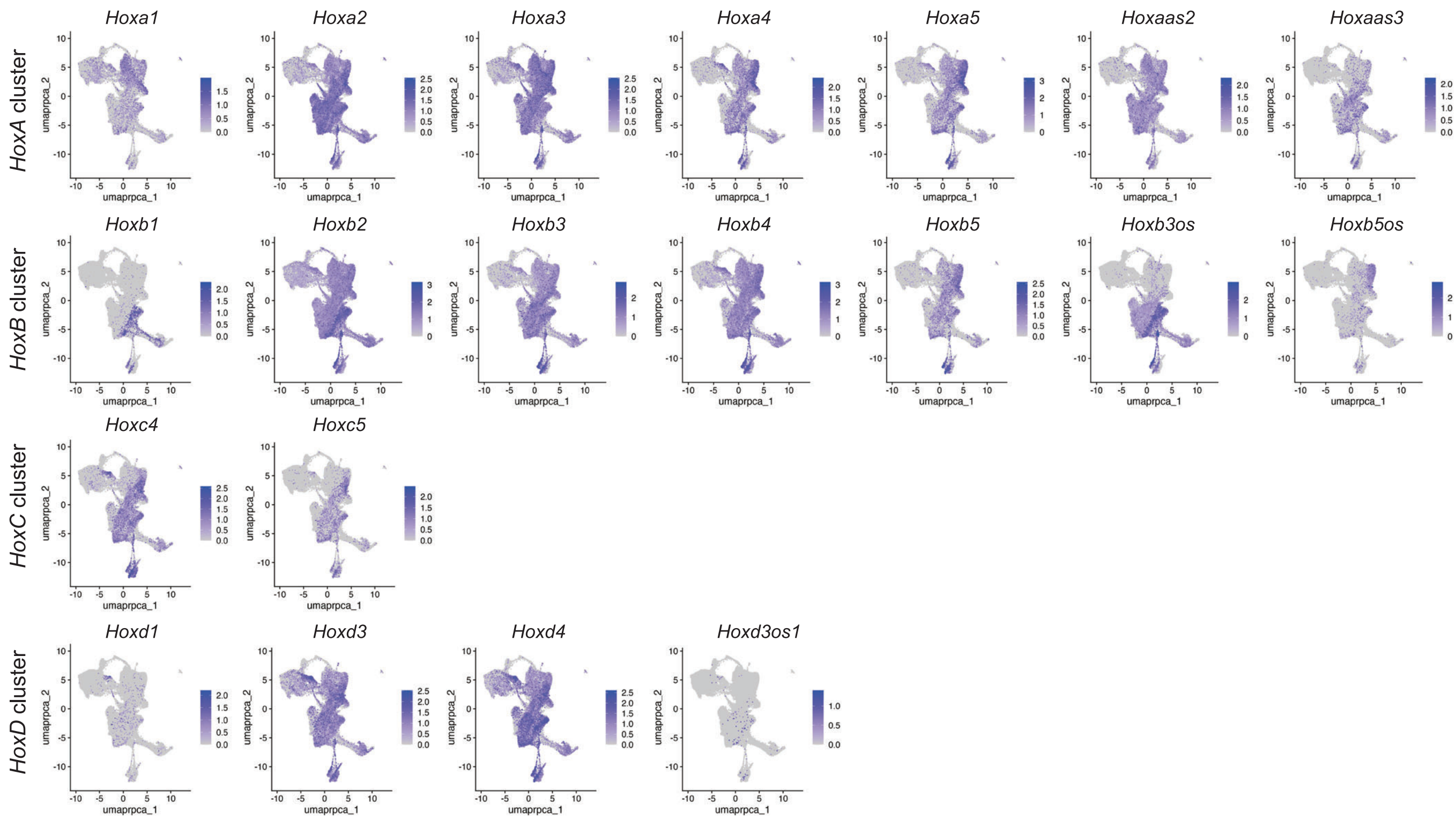

Figure EV8. *Hox* gene expression patterns in NCC-derived lineages.

Feature plots showing the expression of *Hox* genes in NCC-derived lineages.

© The Author(s)

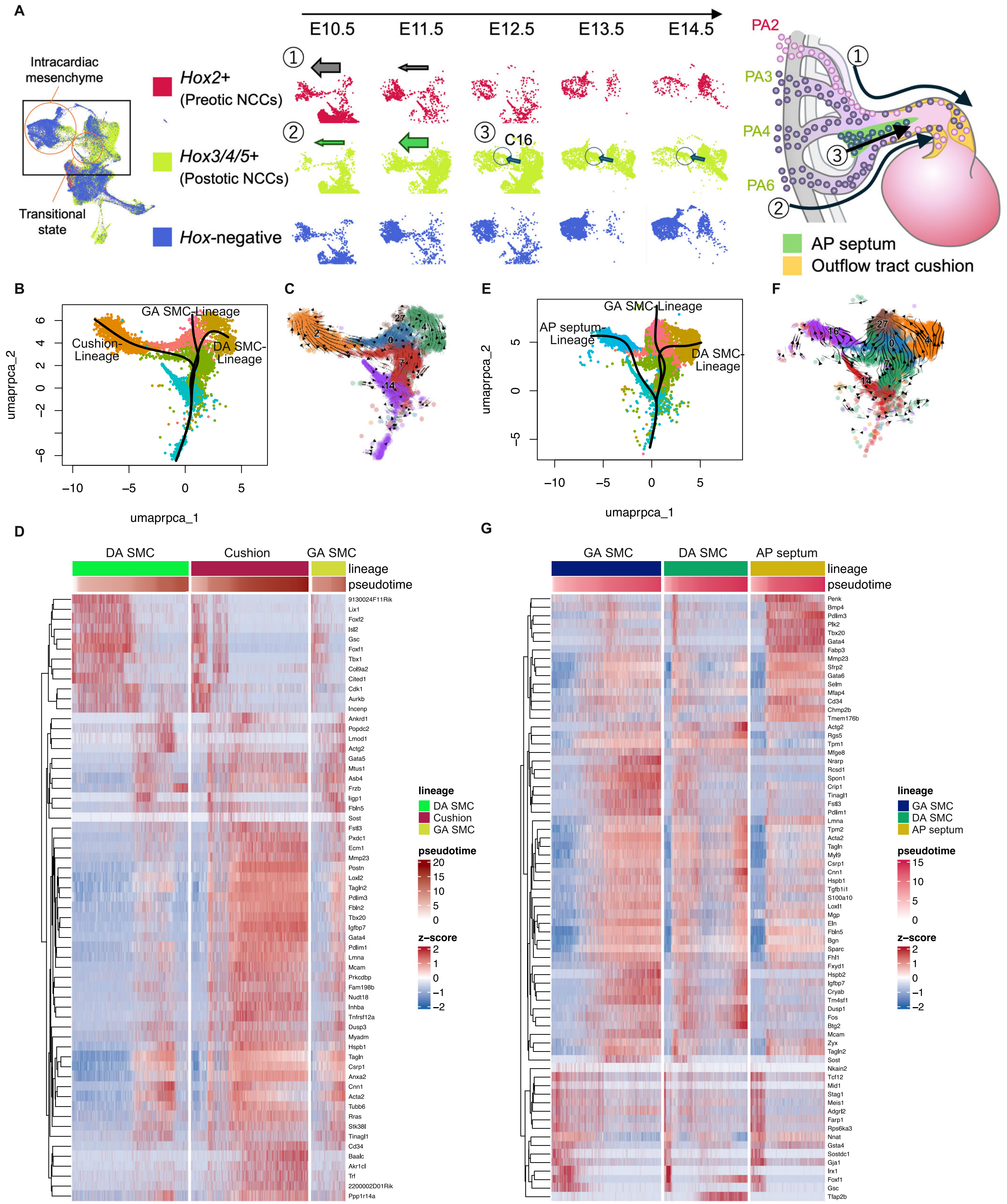
A
E10.5
E11.5
E12.5
E13.5
E14.5
Intracardiac mesenchyme
Transitional state
Hox2+ (Preotic NCCs)
Hox3/4/5+ (Postotic NCCs)
Hox-negative
C16
PA2
PA3
PA4
PA6
AP septum
Outflow tract cushion
B
GA SMC-Lineage
Cushion-Lineage
DA SMC-Lineage
umaprpca_1
umaprpca_2
C
E
AP septum-Lineage
F
D
DA SMC
Cushion
GA SMC
lineage
pseudotime
z-score
G
AP septum

© The Author(s)

Figure EV9. Decoding *Hox* codes in NCC-derived lineages and dynamics of gene expression profiles at the bifurcation point.

(**A**) Schematic illustration of *Hox*-code decoding during development. Cells expressing any *Hox2* paralog, but lacking *Hox3–5* paralogs, were defined as PA2-derived preotic NCCs, whereas cells expressing any of *Hox3*–5 paralogs were classified as PA3/4/6-derived postotic NCCs. Preotic, postotic, and *Hox*-negative populations were then projected onto the integrated UMAP across developmental stages (E10.5–E14.5). Three flows of NCC contribution to the cardiovascular system were inferred across developmental stages. (**B–G**) Trajectory inference based on the Slingshot (**B**, **E**) and scVelo (**C**, **F**) and heatmaps showing gene expression dynamics (**D**, **G**) of NCC-derived lineages at E10.5 and E11.5 (**B–D**) or E12.5, E13.5, and E14.5 (**E–G**). Trajectory was set from C14 to each lineage, and heatmaps showed the top 30 DEGs according to lineages (**D**, **G**). DEGs were calculated as Wald statistics accompanied by pseudotime and filtered with an average log2 expression >1, and the top 30 genes with the result of Wald statistics per cluster were selected. Color scales indicate z-scored values for each gene.

 © The Author(s)

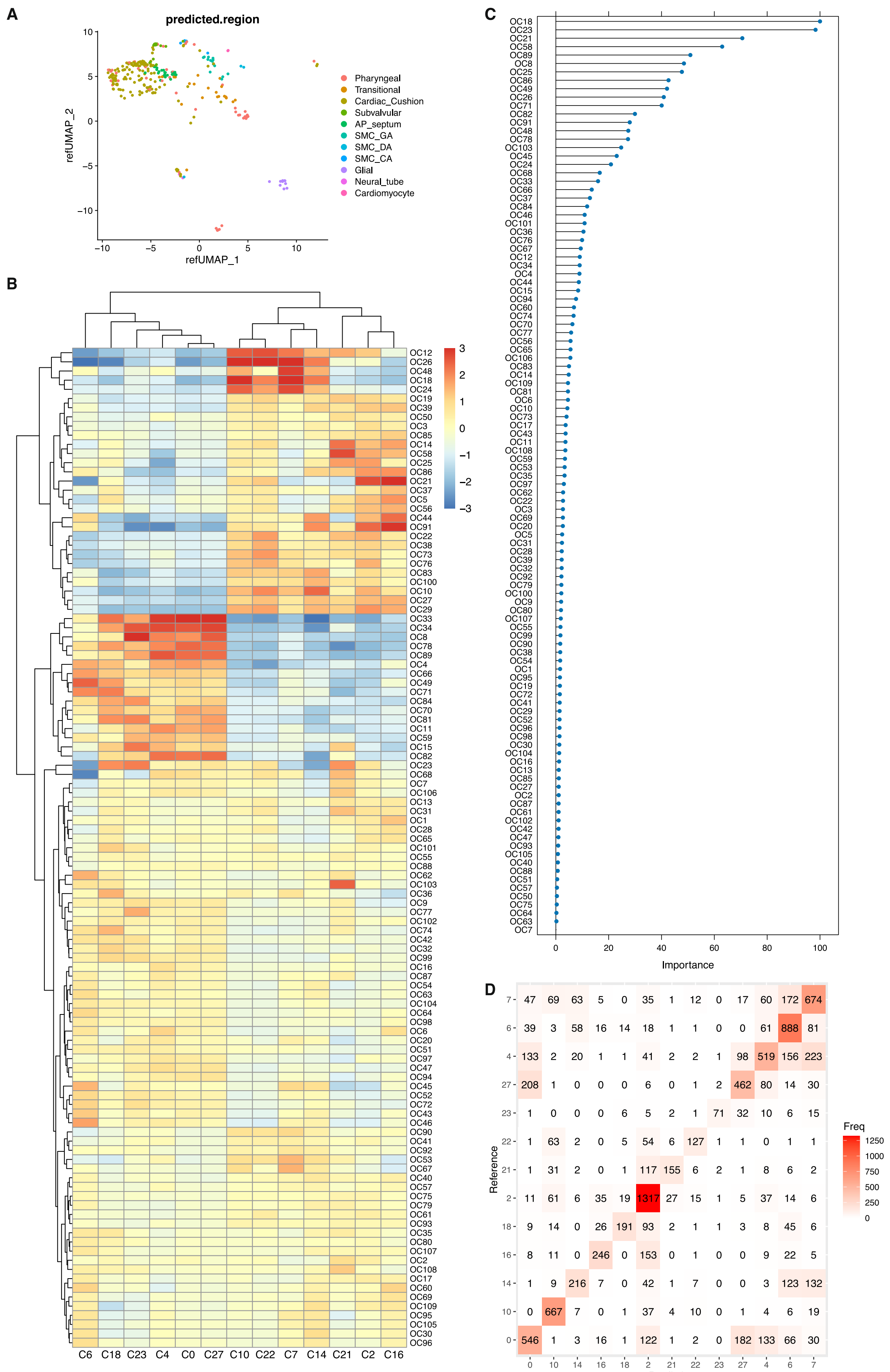
A
predicted.region
Pharyngeal
Transitional
Cardiac_Cushion
Subvalvular
AP_septum
SMC_GA
SMC_DA
SMC_CA
Glial
Neural_tube
Cardiomyocyte
refUMAP_1
refUMAP_2
B
C6 C18 C23 C4 C0 C27 C10 C22 C7 C14 C21 C2 C16
C
Importance
D
Freq
Reference
Prediction

Figure EV10. Characterization of NCC-derived mesenchymal clusters by GRNs represented as overall communities (OCs).

(**A**) Projection of Fluidigm C1 scRNA-seq data onto the integrated UMAP, colored by subcategorization of NCC clusters as shown in Fig. 4A. (**B**) Heatmap showing the enrichment of OCs across integrated mesenchymal clusters. The color scale represents z-scored enrichment values. (**C**) Key OCs regulating NCC lineage commitment were identified using a random forest classifier. (**D**) Confusion matrix showing the performance of the random forest-based model in predicting NCC cluster identity.

 © The Author(s)

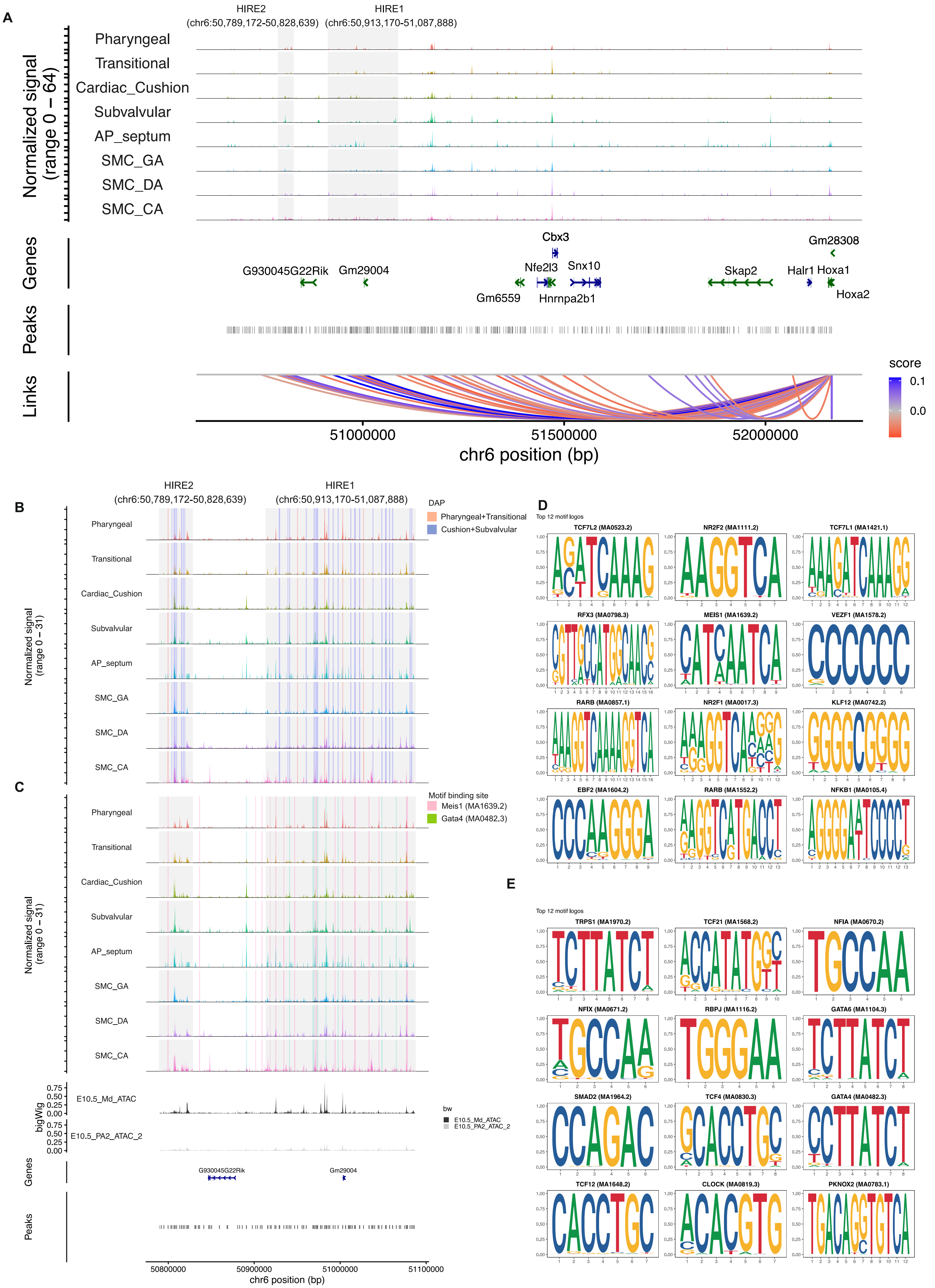
A
HIRE2
(chr6:50,789,172-50,828,639)
HIRE1
(chr6:50,913,170-51,087,888)
Normalized signal
(range 0 – 64)
Pharyngeal
Transitional
Cardiac_Cushion
Subvalvular
AP_septum
SMC_GA
SMC_DA
SMC_CA
Genes
Cbx3
Gm28308
G930045G22Rik
Gm29004
Nfe2l3
Snx10
Skap2
Halr1
Hoxa1
Gm6559
Hnrnpa2b1
Hoxa2
Peaks
Links
score
0.1
0.0
51000000
51500000
52000000
chr6 position (bp)
B
C
D
E
DAP
Pharyngeal+Transitional
Cushion+Subvalvular
Motif binding site
Meis1 (MA1639.2)
Gata4 (MA0482.3)
Normalized signal
(range 0 – 31)
bigWig
E10.5_Md_ATAC
E10.5_PA2_ATAC_2
bw
50800000
50900000
51000000
51100000
Top 12 motif logos
TCF7L2 (MA0523.2)
NR2F2 (MA1111.2)
TCF7L1 (MA1421.1)
RFX3 (MA0798.3)
MEIS1 (MA1639.2)
VEZF1 (MA1578.2)
RARB (MA0857.1)
NR2F1 (MA0017.3)
KLF12 (MA0742.2)
EBF2 (MA1604.2)
RARB (MA1552.2)
NFKB1 (MA0105.4)
TRPS1 (MA1970.2)
TCF21 (MA1568.2)
NFIA (MA0670.2)
NFIX (MA0671.2)
RBPJ (MA1116.2)
GATA6 (MA1104.3)
SMAD2 (MA1964.2)
TCF4 (MA0830.3)
GATA4 (MA0482.3)
TCF12 (MA1648.2)
CLOCK (MA0819.3)
PKNOX2 (MA0783.1)

© The Author(s)

Figure EV11. Chromatin accessibility of *Hoxa2* super-enhancer in NCC-derived mesenchymal clusters.

(**A–C**) Coverage plots of chromatin accessibility at the *Hoxa2* super-enhancer loci (*Hoxa* inter-TAD regulatory element (HIRE) 1 and HIRE2) located 1.33 Mb away from the *Hoxa2* locus in NCC-derived mesenchymal subpopulations. Magnified views of HIRE1(chr6: 50,913,170–51,087,888) and HIRE2 (chr6: 50,789,172–50,828,639) are highlighted in gray (**B**, **C**). Differentially accessible peaks (DAPs) are highlighted in orange (pharyngeal and transitional mesenchyme) and blue (cardiac cushion and subvalvular mesenchyme) (**B**). Transcription factor-binding motifs are indicated in pink (Meis1; MA1639.2) and green (Gata4; MA0482.3). Bigwig tracks of bulk ATAC-seq signals in the mandibular arch (Md) and second pharyngeal arch (PA2) at E10.5. are shown. (**D**, **E**) Top 12 enriched TF-binding motifs identified from DAPs within HIRE1 and HIRE2 in pharyngeal and transitional mesenchyme (**D**) and cardiac cushion and subvalvular mesenchyme (**E**).

 © The Author(s)

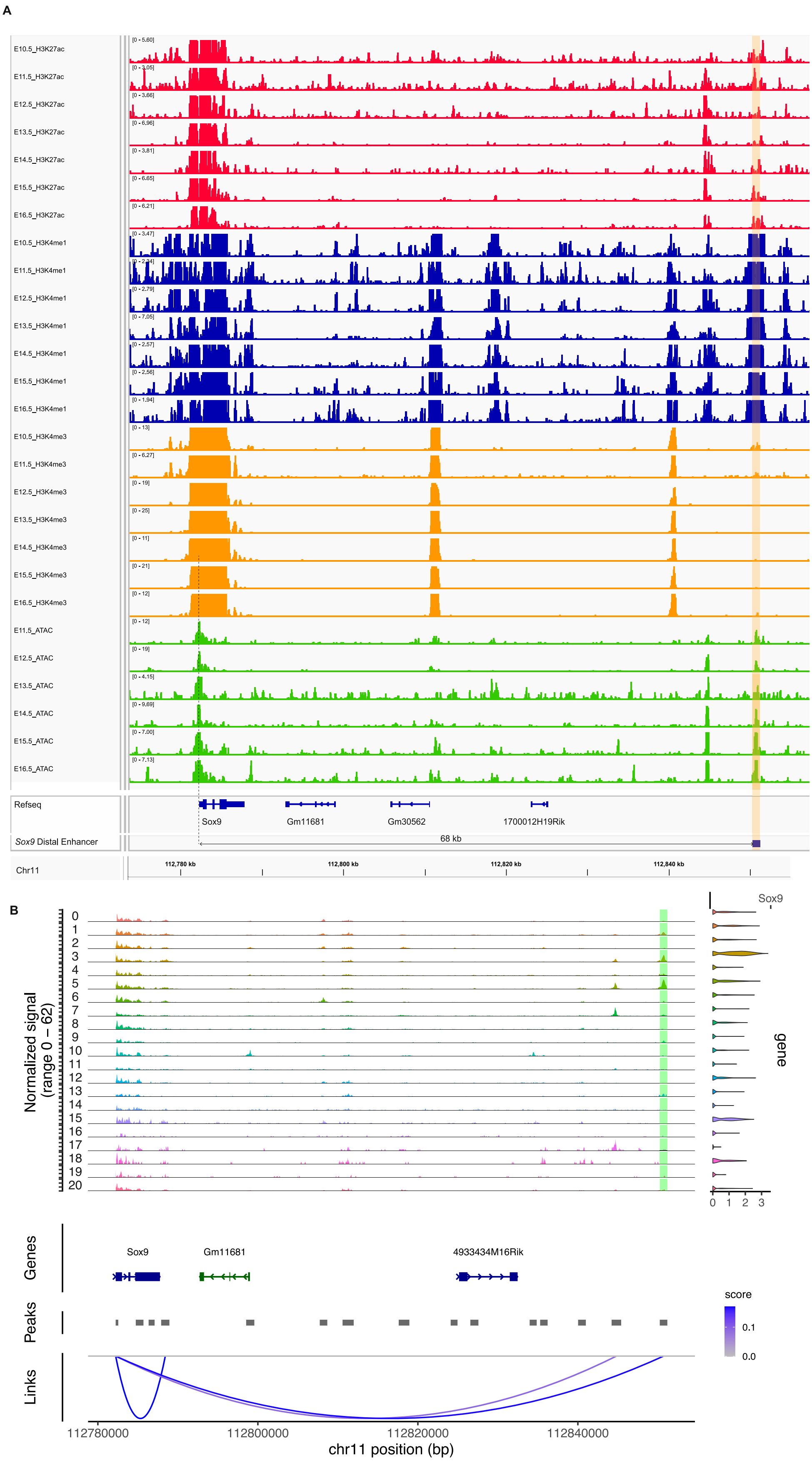
A
E10.5_H3K27ac
E11.5_H3K27ac
E12.5_H3K27ac
E13.5_H3K27ac
E14.5_H3K27ac
E15.5_H3K27ac
E16.5_H3K27ac
E10.5_H3K4me1
E11.5_H3K4me1
E12.5_H3K4me1
E13.5_H3K4me1
E14.5_H3K4me1
E15.5_H3K4me1
E16.5_H3K4me1
E10.5_H3K4me3
E11.5_H3K4me3
E12.5_H3K4me3
E13.5_H3K4me3
E14.5_H3K4me3
E15.5_H3K4me3
E16.5_H3K4me3
E11.5_ATAC
E12.5_ATAC
E13.5_ATAC
E14.5_ATAC
E15.5_ATAC
E16.5_ATAC
Refseq
Sox9
Gm11681
Gm30562
1700012H19Rik
Sox9 Distal Enhancer
68 kb
Chr11
112,780 kb
112,800 kb
112,820 kb
112,840 kb
B
Normalized signal (range 0 – 62)
Sox9
gene
Genes
Sox9
Gm11681
4933434M16Rik
Peaks
Links
score
0.1
0.0
112780000
112800000
112820000
112840000
chr11 position (bp)

Figure EV12. Histone modifications and chromatin accessibility at the *Sox9* locus.

(**A**) ChIP-seq and ATAC-seq data of the E10.5 to E116.5 mouse embryonic heart from ENCODE, accessed via the UCSC Genome Browser (http://genome.ucsc.edu/). The candidate distal enhancer region is highlighted in orange. (**B**) Coverage plots of chromatin accessibility at *Sox9* loci in cardiopharyngeal cell clusters in Fig. 1E (left), with violin plots showing gene expression from scRNA-seq data (upper right). The candidate distal enhancer region is highlighted in light green. Note that Sox9 expression and accessibility to the enhancer region are upregulated in *Tbx20*^{high} mesenchymal (cluster 3) and epicardial (cluster 5) cells.

 © The Author(s)

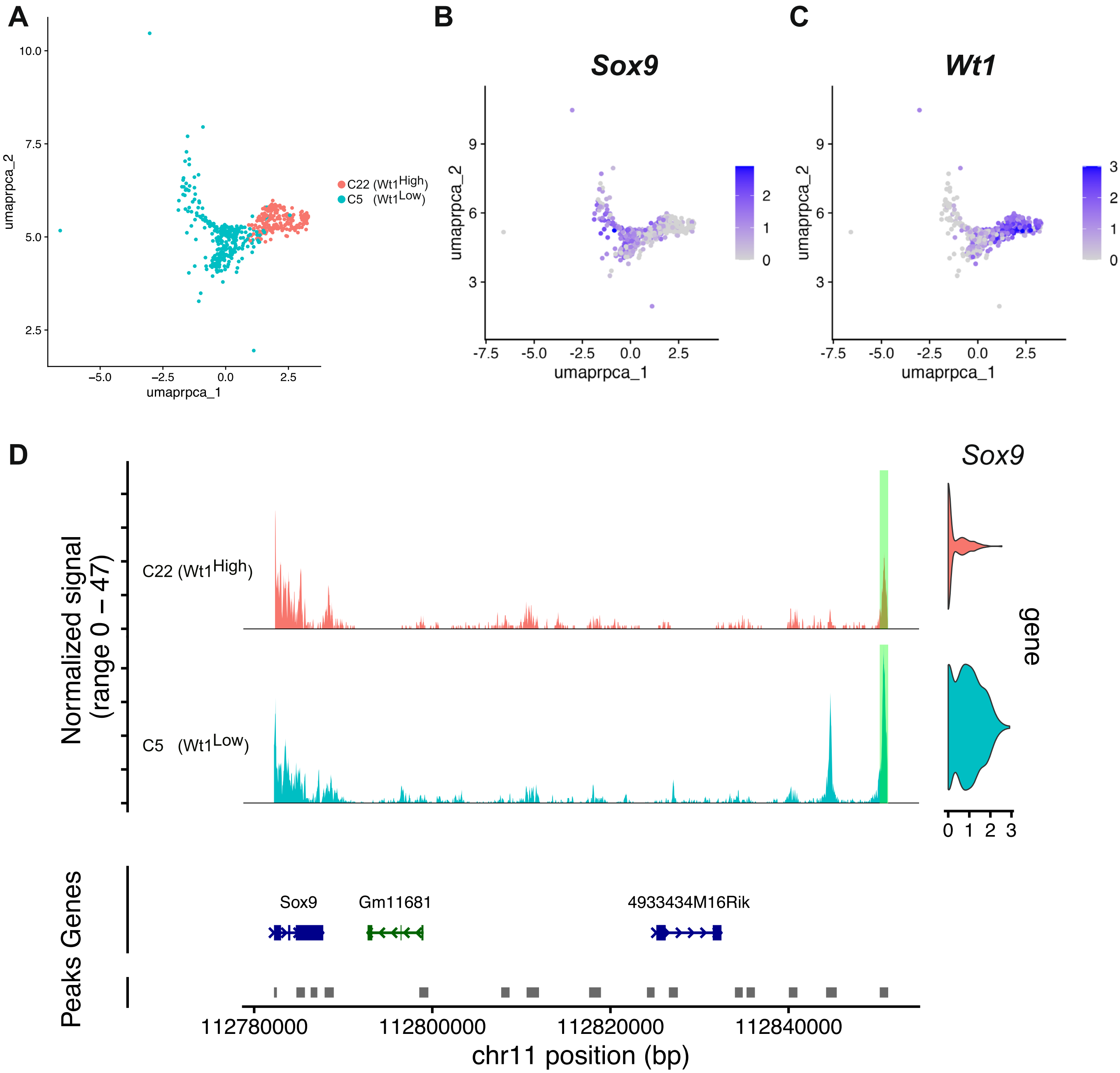

Figure EV13. *Sox9* expression and open chromatin peaks in *Wt1*^high^ and *Wt1*^low^ cell population.

(**A**) UMAP plot of *Wt1*^high^ (C22) and *Wt1*^low^ (C5) clusters extracted from Fig. 1D. (**B, C**) Feature plots showing the expression of *Sox9* (**B**) and *Wt1* (**C**). (**D**) Coverage plot of chromatin accessibility at the *Sox9* locus in *Wt1*^high^ and *Wt1*^low^ cell populations (left), with violin plots showing *Sox9* expression from scRNA-seq data (right).

© The Author(s)
