# Supplementary material for: Hox/Meis-dependent gene-regulatory transition underlies cardiopharyngeal neural crest diversification
Source: EMBO J. 2026 Sep 15;45(19):6978–7020. doi: 10.1038/s44318-026-00886-x (PMC13631262; doi:10.1038/s44318-026-00886-x)
Supplement: Supplementary file 2 [file 44318_2026_886_MOESM1_ESM.pdf]

THE EMBO JOURNAL

Hox/Meis-dependent gene-regulatory transition underlies cardiopharyngeal neural crest diversification

Akiyasu Iwase, Yasunobu Uchijima, Daiki Seya, Mayuko Kida, Hiroki Higashiyama, Kazuhiro Matsui, Akashi Taguchi, Yukihiro Harada, Yunce Wang, Shogo Yamamoto, Shiro Fukuda, Seitaro Nomura, Takahide Kohro, Chisa Shukunami, Haruhiko Akiyama, Masahide Seki, Akinori Kanai, Yutaka Suzuki, Teruhisa Kawamura, Osamu Nakagawa, Hiroto Katoh, Shumpei Ishikawa, Youichiro Wada, Hiroyuki Aburatani, Yukiko Kurihara, Sachiko Miyagawa-Tomita, and Hiroki Kurihara

Corresponding authors: Hiroki Kurihara (kuri-tky@umin.net) , Akiyasu Iwase (akiiwase-tky@umin.ac.jp)

Review Timeline:

Transferred from Review Commons:	3rd Jun 26
Editorial Decision:	7th Jul 26
Revision Received:	15th Jul 26
Accepted:	20th Jul 26

Editor: Ieva Gailite

Transaction Report:

Review COMMONS

This manuscript was transferred to The EMBO Journal following peer review at Review Commons.

(Note: With the exception of the correction of typographical or spelling errors that could be a source of ambiguity, letters and reports are not edited. Depending on transfer agreements, referee reports obtained elsewhere may or may not be included in this compilation. Referee reports are anonymous unless the Referee chooses to sign their reports.)

Review #1

1. Evidence, reproducibility and clarity:

Evidence, reproducibility and clarity (Required)

Iwase et al have used multiomics and spatial transcriptomics to comprehensively map neural crest cell contributions to the mouse heart and great arteries. This careful and detailed analysis reveals changes in the transcriptional profile of neural crest cells as they give rise to different regions and cell types in the heart and great vessels. The study significantly builds on a number of recent scRNA-seq analyses of neural crest cell development and includes development of a new informatic tool for regulatory network investigation. Among the new findings documented are downregulation of Hox gene expression in intracardiac crest cells and regulation of Sox9 by Meis transcription factors. Addressing the following points would improve clarity and accessibility.

1. In Figure 1C it is difficult to visualize all the colors given the mixed contribution of NCC and nonNCC cells to mesenchyme. Please also show YFP transcript distribution in NCC versus nonNCC plots. In addition, it would be helpful to show plots for both NCC and nonNCC for Gata4 and Tbx20.
2. The authors identify a cardiomyocyte cell cluster in their integrated NCC scRNA-seq plots. Are these cells labelled by Wnt1-Cre in the authors' own dataset? Is the trajectory analysis informative as to the steps preceding acquisition of cardiomyocyte fate?
3. Linked with this point, is it possible that there are nonNCC cells in the integrated plots? Of note, many of the NCC genes overlap with genes that have also been shown to be expressed in mesodermal cardiac progenitors (including Osr1, Pparg, Dlk1, Tcf21, Ebf2, Tbx20, Sox9). For example, is it possible to distinguish NCC derived smooth muscle within the heart from cells originating from the second heart field that may express smooth muscle genes? Cluster 27 for example appears broadly expressed in the region of ventricular outlets in Figure 3. Comparison with YFP transcript distribution may be helpful here.
4. Can the authors add any validation of key expression patterns, for example using fluorescent in situ hybridization?
5. Please elaborate on the decoded Hox code patterns that appear to be indicative of arch origins. Do the results allow determination of whether the trajectories to different cardiac fates inferred in Figure 3D differ in different arches?
6. The authors need to explain why the authors place an arrow from mesenchymal cluster 18 to 23 in Figure 3D while the trajectory analysis in 3C predicts the opposite direction.
7. The authors nicely show downregulation of Hox gene expression in NCC cells entering the heart. Can they add discussion of any insights into this from prior studies of loss or gain of Hox gene function?
8. Figure 3Y could be simplified to more clearly distinguish the two types of Meis binding sites. For example, it may be helpful to reorder the mesenchymal cell types based on Hox expression.
9. The authors provide nice in vitro and in vivo evidence for an upstream role of Meis transcription factors in regulating Sox9 expression. Can the authors identify from the enhancer sequence (or their transcriptomic dataset) any of the non-Hox transcription factors that Meis may

be working with here? Please discuss the significance of Sox9 expression in epicardium driven by the same enhancer. Might this regulation also operate in second heart field progenitor cells where both genes are expressed? It is not evident in Figure 7 that Sox9-EGFP is also expressed in epicardium.
10. Could this approach yield similar data for Osr1? Please clarify if there is any experimental evidence supporting the predicted negative regulation of Sox9 by Osr1 in the heart illustrated in Figure 8.
11. Concerning the links between valve mesenchyme and skeletogenic programs it would be relevant to cite the earlier work of Lincoln and Yutzey (reviewed in PMID: 16643886):
12. In order to increase accesibility of the dataset the authors are encouraged to include a browser link.

Minor points:

13. The authors could rephrase the title since the term topographical genetic switch is unclear.
14. In the introduction, with reference to the De Bono study, please note that Tbx1 was shown to regulate pharyngeal NCC differentiation stage transitions non-cell autonomously.

2. Significance:

Significance (Required)

Iwase et al have used multiomics and spatial transcriptomics to comprehensively map neural crest cell contributions to the mouse heart and great arteries. This careful and detailed analysis reveals changes in the transcriptional profile of neural crest cells as they give rise to different regions and cell types in the heart and great vessels. The study significantly builds on a number of recent scRNA-seq analyses of neural crest cell development and includes development of a new informatic tool for regulatory network investigation. Among the new findings documented are downregulation of Hox gene expression in intracardiac crest cells and regulation of Sox9 by Meis transcription factors.

3. How much time do you estimate the authors will need to complete the suggested revisions:

Estimated time to Complete Revisions (Required)

(Decision Recommendation)

Between 1 and 3 months

4. *Review Commons* values the work of reviewers and encourages them to get credit for their work. Select 'Yes' below to register your reviewing activity at [Web of Science]

Reviewer Recognition Service (formerly Publons); note that the content of your review will not be visible on Web of Science.

Web of Science Reviewer Recognition

No

Review #2

1. Evidence, reproducibility and clarity:

Evidence, reproducibility and clarity (Required)

Summary

In this manuscript, Iwase et al. cleverly make use of different modalities, spatial transcriptomics and single-cell omics datasets, in conjunction with a well-established Wnt1-Cre;R26R-EYFP line to trace neural crest cells (NCCs) contributing to the cardiovascular system during embryonic development in the mouse.
By doing so, the authors identified a bifurcation between cardiac NCCs contributing to the OFT cushions and forming the aorticopulmonary (AP) septation complex. Thus, the authors split "intracardiac NCCs" into two different NCC programs/compartments, even though both reside in the broad OFT region. The NCCs that enter and associate with the OFT cushions undergo a Hox off transition (Hox-positive to Hox-negative once intracardiac), with a corresponding shift in Meis binding and GRN wiring. The authors propose these cells pass through a Meis2-Sox9-Scx "skeletogenic progenitor-like" intermediate and contribute to semilunar valves and coronary artery smooth muscle. By contrast, the NCCs assigned to aorticopulmonary septum (APS) formation and great vessel smooth muscle retain a distinct Hox codes.

Major Comments

1. The manuscript would benefit from clearer delineation between the different NCC contributions, particularly for non-specialist readers. The distinction between (i) CNCCs in OFT cushions and (ii) CNCCs forming the aorticopulmonary (AP) septation complex is not adequately explained. While both populations contribute to OFT septation, according to the authors, they represent distinct compartments with different developmental trajectories. The authors could clarify this using anatomically labelled hearts at the stages they conduct their analysis, along with additional text and schematics explaining what is meant by each compartment. This would greatly enhance the accessibility of the manuscript.
2. The Methods state that "Decomposition of cell clusters of scRNA-seq was performed by RCTD to map them onto the Xenium dataset," but this description is insufficient. The authors

should clarify whether RCTD was applied separately for each developmental stage (i.e., E11.5 scRNA-seq reference for E11.5 Xenium, E12.5 reference for E12.5 Xenium), or whether a pooled reference was used across stages. This clarification is important because RCTD performance depends critically on the correspondence between reference and target datasets. Using a multi-stage integrated reference to deconvolve stage-specific spatial data could introduce artifacts, as cell states and cluster compositions vary considerably across developmental timepoints.

3. Figure 2j-m needs annotations and shcematics. It is currently very difficult to identify the different compartments. See Figure C in Chen et al. for an example of this approach. In addition, what are the fine clusters from 1 to 20? Which ones are NCC-derived?

4. The panels show pharyngeal markers, OFT/intracardiac markers, and SMC markers in Figures 2n-y, but could the authors show the proportion of NCC-derived (YFP+) cells for each cluster? Could the authors also map only the YFP+ cells on the Xenium data? It would be useful to see the proportion of YFP-positive (NCC-derived) cells for each delineated compartment. YFP-positive cells appear to exist at the boundary between LV and RV in the septum, this observation would benefit from proper quantification.

5. Figure 3 is confusing because it integrates data from multiple overlapping stages (E8.5 to P7). While the authors identify distinct compartments, pharyngeal mesenchyme, intracardiac mesenchyme, and SMCs, it is unclear why stages beyond E14.5 and E17.5 (corresponding to the initial single-cell omics and Visium analysis) were included. Although leveraging additional datasets is a clever approach, the integration of data from such disparate developmental timepoints confounds interpretation. For example, Cluster 6 appears to include cells from both P7 and E12.5 stages. Given that the Visium data represent hearts at E14.5 and E17.5, it is problematic to map clusters derived from other stages onto these spatial datasets.

6. Throughout the manuscript, the authors describe "lineage relationships" between cell populations, but these are in fact developmental trajectories inferred computationally (via UMAP connectivity and RNA velocity), not true lineage relationships. This distinction is critical and should be explicitly stated.

For example, the statement "we propose that intracardiac NCCs within C10 and C22 differentiate via C2 into valvular (C21) and subvalvular (C18) interstitial cells" should be framed as a computational inference, not an established lineage relationship. Without clonal lineage tracing data, these claims cannot be verified.

Similarly, the claim that "NCCs contributing to the AP septum are distinct from other intracardiac NCCs in that they do not populate the cardiac cushions but remain continuous with NCCs populating the distal outflow tract cushion" lacks direct lineage evidence. What experimental data support this assertion?

In addition, the authors state that what De Bono et al. identified as "outflow smooth muscle" corresponds in their dataset to early intracardiac mesenchymal clusters C2, C10, and C22, present as early as E10.5, when mesenchymal NCC derivatives express immature SMC markers, and that these cells later differentiate into coronary artery SMCs around E14.5 (C23) as well as other non-muscle components. This claim is not verified, whether these cells are indeed the ones differentiating into coronary artery SMCs is based solely on computational inference from C2, C10, C22 to C23.

7. Claims regarding marker expression in specific compartments (for exmaple Hapln1 and Postn in cushions) require additional spatial validation at higher resolution than what is currently provided by the Visium data. Moreover, it is unclear whether these data are single-cell resolution; the authors need to clarify this. HCR staining would be ideal to confirm these expression patterns. Currently, all conclusions are based solely on gene expression without orthogonal spatial confirmation. At minimum, the authors should provide references from the literature supporting these expression patterns.
8. Could the authors confirm the absence of the Sox9high/Scxhigh population in AP septum descendants?

Minor Comments

Could the authors better justify their choice of stages (E11.5 to E17.5) for the single-cell multiomic assay? Given that OFT cushions are already populated by NCCs by E10.5 and that AP septum formation is already underway at this stage (see Development (2007) 134(8): 1593-1604), the rationale for beginning at E11.5 should be explicitly stated.
Spelling errors in Figure 2e: "ambious" should be "ambiguous"; "atrium venticle" should be "atrium/ventricle"; "ventricle" is misspelled in several locations.
The clustering resolution is very high, yielding many clusters that are difficult to distinguish based on the colour code alone. What does "ventricle_CL" refer to? What is the "marginal layer"? A clearer legend or supplementary table defining each cluster would be helpful.
Figure 2j: The image is very dim.
Figure 3b: What do the numbers correspond to? Additionally, "mesenchyme" is misspelled.

Referees cross-commenting

I also agree with the other reviewers' comments. Many thanks.

2. Significance:

Significance (Required)

The overall approach is sound and the datasets generated are valuable resources for the field. The manuscript presents interesting findings regarding NCC heterogeneity in cardiac development.

As I understand the authors' model:
OFT cushion-associated NCCs enter and associate with the OFT cushions, undergoing a Hox-positive to Hox-negative transition, accompanied by a shift in Meis binding and GRN rewiring. These cells pass through a Meis2-Sox9-Scx "skeletogenic progenitor-like" intermediate state and contribute to semilunar valves and coronary artery smooth muscle.
Aorticopulmonary septum NCCs, by contrast, retain distinct Hox codes (Hox4/5 enrichment) rather than becoming Hox-negative, and contribute to the AP septum and great vessel smooth muscle.

Strengths: The integration of multiple omics modalities with lineage tracing is a powerful approach, and the identification of a Hox-dependent bifurcation in NCC fate is a novel

conceptual advance.

Limitations: The reliance on computational trajectory inference without orthogonal lineage validation, combined with the integration of datasets spanning very different developmental stages, limits the strength of some conclusions. The analysis also required more precise anatomical annotations to facilitate accessibility to the readers - to visualise better the distinguishable contribution of the cardiac NCCs to the OFT.

Advance

The study extends knowledge in the field by providing novel mechanistic insight into neural crest diversification in the context of cardiovascular development. The nature of the advance is primarily mechanistic, identifying a Hox-Meis regulatory switch that distinguishes cushion-associated from cushion-independent NCC lineages.

Audience

This work will be of interest to a specialised audience interested in neural crest cells and developmental biologists using omics approaches to address cell fate diversification in complex tissues.

Reviewer Expertise

Developmental biology, lineage analysis, mouse genetics. I do not have the expertise to assess the computational methods used in this paper.

3. How much time do you estimate the authors will need to complete the suggested revisions:

Estimated time to Complete Revisions (Required)

(Decision Recommendation)

Between 3 and 6 months

4. *Review Commons* values the work of reviewers and encourages them to get credit for their work. Select 'Yes' below to register your reviewing activity at [Web of Science Reviewer Recognition Service] (formerly Publons); note that the content of your review will not be visible on Web of Science.

Web of Science Reviewer Recognition

Yes

Review #3

1. Evidence, reproducibility and clarity:

Evidence, reproducibility and clarity (Required)

Summary:

Iwase et al. presents a comprehensive multiomics analyses of cardiac neural crest cell (NCC) differentiation during cardiopharyngeal development. Using Wnt1Cre;R26R-EYFP mice, the authors isolated NCCs and non-NCCs at E11.5, E12.5, E14.5 and E17.5 stages and performed single-cell RNA-seq, ATAC-seq, spatial transcriptomics analyses. Spatial resolution of NCC-derived populations was achieved using Xenium (E11.5-E12.5) and Visium (E14.5-E17.5) platforms. Integration of single cell and spatial datasets identified distinc NCC-derived populations with defined spatial organization within the pharyngeal and intracardiac regions. The study concludes that Hox gene patterning underlies NCC subpopulation identity during cardiopharyngeal development and reveals a developmental transition from Hox-dependent to Hox-independent transcriptional regulation. Furthermore, the authors identify a Meis2-Sox9-Scx gene regulatory network making a skeletogenic progenitor-like intermediate that contribute to coronary smooth muscle and semilunar valve formation.

While the dataset is comprehensive and technically strong, several key conclusions are not always convincingly supported by enough data. As a result, some claims appear speculative and would benefit from additional experimental validation to strengthen the proposed developmental models.

I would strongly encourage authors to consider the following points to provide additional details that will strengthen their study:

Major comments:

- The authors should provide detailed FACS gating strategies and sorting conditions used to selectively isolate EYP-positive and EYP-negative NCC populations, including representative plots and information on exclusion criteria (e.g., doublets, dead cells).
- Although the authors isolated nuclei for both scRNA-seq and ATAC-seq, the number of cardiomyocytes within the EYPF-negative population is unexpectedly low. The authors should clarify potential technical or biological reasons for this underrepresentation (e.g., nuclei isolation efficiency, sorting strategy, filtering criteria, or developmental stage-specific composition).
- In figure 1, the authors present results from unsupervised clustering of 9,420 cells into 21

distinct clusters, many of which are broadly labeled as "mesenchymal cells". The authors should refine this nomenclature by providing more specific annotations or defining criteria, as this broad classification limits interpretability of the identified subpopulations.
- To integrate spatial annotated Xenium datasets with scRNA-seq data, the authors used Tangram, enabling estimation of the spatial distribution of EYFP-positive NCCs within the pharyngeal region of E11.5 and 12.5 embryos. However, the E11.5 section show a relatively low number of EYFP-positive cells (Figure 2f). The authors should clarify whether this reflects technical limitations (e.g., probe design, segmentation efficiency, or integration parameters) or biological factors and explain how this affects interpretation of the spatial analyses.
- The author used RCTD tool to decompose the scRNA-seq dataset into NCC and non-NCC components and mapped these onto the Xenium dataset. However, panels j and l in Figure 2 show low signal in the E11.5 sections. The authors should clarify whether this reflects technical limitations of the RCTD deconvolution, differences in sampling, or biological factors, and discuss how this result impacts of interpretation of the spatial mapping results at this stage.
- The authors integrated their data with publicly available scRNAs-eq datasets of NCCs from E8.5 to P7 hearts and present results from unsupervised clustering of 67,208 cells into 28 distinct clusters. Figures 3a and 3b show that cardiomyocyte (C26) is included in NCC-derivatives. The authors should clarify whether this reflects technical issue when they made FACS.
- The authors used RNA-velocity to infer relationship among the identified clusters. However, this analysis requires particular caution given that data were generated from multiple datasets obtained under different conditions. Several conclusions drawn from the RNA-velocity analysis are not convincing, as illustrated in Figures 3c and 3d, where the inferred velocity directions appear inconsistent with the proposed developmental model (e.g., trajectory from cluster 23 toward 18 or from 4 toward 6). The authors should clarify these discrepancies, justify the integration of heterogenous datasets and reassess the interpretation of the inferred lineage relationships.
- The authors should provide more detail on how they identified bifurcation points and more clearly explain the transition from intracardiac mesenchyme to smooth muscle cells (SMC). Additionally, they should clarify what distinguishes the three clusters (C4, C23, C27) in terms of transcription programs, marker expression, or functional states, to better support their proposed differentiation trajectories.

Minor comments:

- The authors convincingly demonstrate a switch in Meis-binding motifs across NCC populations, supporting a model in which cardiac cushion-associated NCCs transition from Hox-dependent to Hox-independent transcriptional regulation via alternative cofactor interactions and DNA-binding preferences. However, the authors should provide evident on whether GATA motifs are enriched within Meis peaks, as this could further clarify potential cooperative interactions during this transition.
- In Figure 5 panels g, j and k are difficult to interpret. The authors should provide clearer annotations, labeling, or additional explanations to improve readability and facilitate understanding of the data.
- In Figure 6, results support the role of hexameric Meis-binding motif-containing region as a distal enhancer of Sox9. The authors should provide additional results from a ChiP-qPCR

experiment to further validate this model.
- Panel l in Figure S3 requires better annotation.
- Correct the typo errors in Figure 5a.
- The authors should refer to previous studies showing the role of Hoxa1 and Hoxb1 in the development of great arteries or semilunar valves.

Referees cross-commenting

Having read the comments of the other reviewers, I totally agree with them. All our comments converge and should allow the authors to improve their manuscript.

2. Significance:

Significance (Required)

The study provides high-resolution spatial and temporal mapping of NCC-derived populations and proposes mechanistic insights into Hox-dependent versus Hox-independent transcriptional regulation, as well as a Meis2-Sox9-Scx gene regulatory network contributing to smooth muscle and semilunar valve formation.

Strengths and limitations: The datasets are rich and well-integrated, offering valuable resources for the field. However, several key conclusions rely on correlative analyses and heterogeneous datasets, making some claims speculative. Technical details, such as FACS gating, low representation of cardiomyocytes, and interpretation of RNA velocity, require further clarification, which currently limits the strength of the mechanistic inferences.

Advance: This work advances the understanding of NCC lineage diversification and gene regulatory dynamics in cardiopharyngeal development, particularly highlighting potential transcriptional switches and intermediate progenitor states that guide structural formation in the heart.

Audience: The study will be of interest to researchers in developmental biology, cardiovascular biology, and single-cell multi-omics, particularly those studying neural crest cell differentiation and cardiac morphogenesis.

3. How much time do you estimate the authors will need to complete the suggested revisions:

Estimated time to Complete Revisions (Required)

(Decision Recommendation)

Between 3 and 6 months

4. *Review Commons* values the work of reviewers and encourages them to get credit for their work. Select 'Yes' below to register your reviewing activity at Web of Science Reviewer Recognition Service (formerly Publons); note that the content of your review will not be visible on Web of Science.

Web of Science Reviewer Recognition

Yes

Responses to the reviewers

First of all, we would like to thank all the reviewers for their valuable and constructive comments on our manuscript. We have considered each comment and revised the manuscript accordingly. We respond to each comment below in **blue font**.

To Reviewer #1

Reviewer #1 (Evidence, reproducibility and clarity (Required)):

Iwase et al have used multiomics and spatial transcriptomics to comprehensively map neural crest cell contributions to the mouse heart and great arteries. This careful and detailed analysis reveals changes in the transcriptional profile of neural crest cells as they give rise to different regions and cell types in the heart and great vessels. The study significantly builds on a number of recent scRNA-seq analyses of neural crest cell development and includes development of a new informatic tool for regulatory network investigation. Among the new findings documented are downregulation of Hox gene expression in intracardiac crest cells and regulation of Sox9 by Meis transcription factors. Addressing the following points would improve clarity and accessibility.

Thank you for your encouraging feedback and comments. We have responded to your comments below.

1. In Figure 1C it is difficult to visualize all the colors given the mixed contribution of NCC and nonNCC cells to mesenchyme. Please also show YFP transcript distribution in NCC versus nonNCC plots. In addition, it would be helpful to show plots for both NCC and nonNCC for Gata4 and Tbx20.

To improve visualization, we separated EYFP-positive NCCs and EYFP-negative non-NCCs into distinct plots (Figure 1e), rather than displaying EYFP transcript distribution within a single combined plot. In addition, we have included separate plots for both NCCs and non-NCCs showing the expression of *Gata4* and *Tbx20* (Figure 1g, h).

Furthermore, in the revised manuscript, we subdivided the original clusters c5 and c13 into two subclusters each, resulting in a total of 23 clusters in the UMAP shown in Figure 1. This

refinement was introduced to facilitate clearer interpretation and subsequent analyses.

2. The authors identify a cardiomyocyte cell cluster in their integrated NCC scRNA-seq plots. Are these cells labelled by Wnt1-Cre in the authors' own dataset? Is the trajectory analysis informative as to the steps preceding acquisition of cardiomyocyte fate?

A total of 20 EYFP-positive NCCs in our own dataset were assigned to the cardiomyocyte cluster in the integrated UMAP. Of these, 6 cells were located within the cardiomyocyte cluster (c21), while the remaining cells were classified as pharyngeal mesenchyme but positioned in close proximity to c21 in the original UMAP (Figure 1e). Although this observation is consistent with previous reports showing that NCCs give rise to cardiomyocytes, the small number of cells precluded meaningful characterization or trajectory analysis of cardiomyocyte fate acquisition. Accordingly, we have addressed this point only briefly in the revised manuscript as follows:

Only a few NCCs were detected within cardiomyocyte clusters, which were predominantly composed of non-NCCs, consistent with previous reports demonstrating NCC differentiation into cardiomyocytes[15,16]. The overall number of cardiomyocytes was low, likely reflecting the restricted sampling of the cardiac outflow tract (Figure 1a).
(P6, L20–25)

3. Linked with this point, is it possible that there are nonNCC cells in the integrated plots? Of note, many of the NCC genes overlap with genes that have also been shown to be expressed in mesodermal cardiac progenitors (including Osr1, Pparg, Dlk1, Tcf21, Ebf2, Tbx20, Sox9). For example, is it possible to distinguish NCC derived smooth muscle within the heart from cells originating from the second heart field that may express smooth muscle genes? Cluster 27 for example appears broadly expressed in the region of ventricular outlets in Figure 3. Comparison with YFP transcript distribution may be helpful here.

In principle, non-NCCs were excluded from the integrated plots shown in Figure 3. However, we cannot completely rule out the possibility that a small number of non-NCC cells were inadvertently included, for example due to false-positive signals during cell isolation. In our dataset, NCCs and non-NCCs were stringently distinguished based on FACS profiles, detection of EYFP transcript reads in the RNA-seq data, and rigorous exclusion of doublets and low-quality

cells.

Regarding the distinction between NCC- and non-NCC-derived SMCs, a key challenge lies in defining comparable cell populations. We first validated the annotations of SMC clusters (C4, C23, and C27) using immunostaining for Myh11, Sost, and Reln, confirming consistency with their transcriptomic identities. We then re-clustered the SMC populations and projected non-NCC SMCs (clusters 4 and 20 in Figure 1d) onto this UMAP. These non-NCC SMCs were mapped to clusters corresponding to great artery and coronary artery SMCs (C27 and C23, respectively).

However, we lack confidence that these projected populations are directly comparable. For example, non-NCC SMCs mapped to C23 or C27 may not necessarily represent bona fide coronary or great artery SMCs from equivalent anatomical regions, and could include other SMC subtypes such as venous SMCs or pericytes. Given the known regional heterogeneity of SMCs and the absence of strict spatial matching criteria, such comparisons would be difficult to interpret. This limitation is further compounded by the relatively small number of cells available.

For these reasons, we focused on spatial validation of cluster annotations by immunostaining in this study, and have reserved detailed comparisons between NCC- and non-NCC-derived SMCs for future work. We believe this does not detract from the overall consistency or value of the present study.

4. Can the authors add any validation of key expression patterns, for example using fluorescent in situ hybridization?

Figures 2n-y present Xenium-based multiplexed fluorescent in situ hybridization data that validate the spatial expression patterns of marker genes characterizing NCC derivatives in pharyngeal mesenchyme, intracardiac mesenchyme, and SMC populations. In addition, we have incorporated new Xenium images highlighting key gene expression patterns in the aorticopulmonary (AP) septum at E12.5 (Figure S4), supporting the annotation of cluster C16 in the integrated UMAP as corresponding primarily to the AP septum. We have also added immunostaining data for Myh11, Sost, and Reln to further validate the annotations of SMC clusters (see the response above). Together, these data provide independent spatial confirmation of the transcriptional signatures identified in our single-cell analyses. Based on these data, we revised the relevant section of the Results as follows:

The SMC clusters, which were continuous with the pharyngeal mesenchyme via transitional

populations in the UMAP, were identified by high expression of the mature SMC marker Myh11 (Figure 3i). Differential gene expression analysis further distinguished individual clusters (Figure S4a-g). Among these, C27 displayed a transcriptomic profile characteristic of the great artery SMCs, including high expression of Sost (Figure S3j). C4 was enriched for Tfap2b and Ptger4 (Figure S3j), markers of the ductus arteriosus SMCs[21,22], supporting its annotation. C0 and C7 likely represent transitional states between pharyngeal mesenchyme and differentiated lineages, potentially bifurcating toward great artery SMCs or cardiac cushion mesenchyme (Figure S3a and Table S6). C23 was characterized by high expression of Gja4, a marker of coronary artery SMCs, along with pericyte markers Kcnj8 and Rgs5 (Figure 3j and Figure S3k), corresponding to the cluster similarly annotated by Chen et al[14]. In addition, C23 was also distinguished from C4 and C27 by its expression of Reln (Figure S4d).

Immunostaining supported these cluster annotations. Sost expression is observed in great artery SMCs but not in ductus arteriosus and coronary artery SMCs, whereas Myh11 expression was higher in ductus arteriosus and coronary artery SMCs than in aortic SMCs (Figure S4f-m). Furthermore, Reln expression was restricted to coronary artery SMCs (Figure S4n-s).

(P8, L30 – P9, L14)

5. Please elaborate on the decoded Hox code patterns that appear to be indicative of arch origins. Do the results allow determination of whether the trajectories to different cardiac fates inferred in Figure 3D differ in different arches?

In response to the reviewer's suggestion, we have further elaborated on the decoded *Hox* code patterns indicative of pharyngeal arch origin and examined whether trajectories toward distinct cardiac fates differ between arch-derived NCC populations (see new supplemental figure).

To further delineate Hox code patterns associated with pharyngeal arch origin, we stratified the integrated UMAP by distinct Hox expression profiles (Figure S9). Cells expressing any Hox2 paralog, but lacking Hox3–5 paralogs, were defined as PA2-derived preotic NCCs, whereas cells expressing any of Hox3–5 paralogs were classified as PA3/4/6-derived postotic NCCs. Preotic, postotic, and Hox-negative populations were then projected onto the integrated UMAP across developmental stages (E10.5–E14.5). Trajectory inference indicated that transitions toward intracardiac mesenchyme occur earlier in preotic cells (E10.5) than in postotic cells (E11.5), consistent with their known sequential migration into the cardiac cushion[8]. From

E12.5 to E14.5, postotic cells showed a progressive emergence of the aorticopulmonary septum–associated cluster C16 from transitional states. Notably, the proportion of Hox-negative cells increased within intracardiac mesenchyme, except in C16 where Hox expression was retained, supporting the notion that Hox genes are broadly downregulated in cushion-associated intracardiac NCCs (Figure 4k, S9).

(P11, L24 – P12, L4)

6. The authors need to explain why the authors place an arrow from mesenchymal cluster 18 to 23 in Figure 3D while the trajectory analysis in 3C predicts the opposite direction.

RNA velocity analysis of scRNA-seq data is fundamentally based on splicing dynamics. The original framework assumes that transcriptional induction and repression persist long enough for cells to reach active (transcribing) or inactive (silenced) steady-state equilibrium. However, this assumption is often violated during cell differentiation, where transient cell populations frequently exhibit rapidly changing mRNA levels that do not reach steady-state equilibrium. To address this limitation, the scVelo method was developed (Bergen et al., 2020, *Nature Biotechnology*), and we applied this approach to the integrated NCC datasets in the present study. This analysis successfully inferred directional flows from the pharyngeal mesenchyme toward SMCs and intracardiac mesenchyme through transitional states (Figure 3c,d). However, as the reviewer correctly pointed out, the analysis predicted a directional flow from C23 to C18, apparently opposite to the biological directionality supported by previous findings (see P9, L15–19).

We consider this discrepancy to reflect intrinsic limitations of RNA velocity analysis. As discussed by Bergen et al., in systems containing multiple lineages and cellular processes, differences in gene regulatory networks among heterogeneous cell states can generate multiple trajectories in phase space owing to distinct splicing kinetics. In addition, incompletely captured splicing kinetics may represent only a limited portion of the overall dynamics, particularly near terminal differentiation states. In such cases, phase portraits of unspliced versus spliced transcripts may appear nearly linear rather than curved, potentially leading to erroneous assignment of positive or negative RNA velocities. Consistent with this limitation, we obtained opposite directionalities between C18 and C23 depending on whether steady-state or dynamical models were applied and according to different parameter settings. Through these repeated computational re-evaluations of lineage directionality, we concluded that RNA velocity analysis is suitable for

capturing the global landscape of differentiation flow, but that accurate inference of local lineage directionality may require careful model selection and parameter optimization to ensure consistency with established biological evidence.

To avoid arbitrariness and potential confusion, we removed the arrow between C18 and C23 from the revised Figure 3d. Instead, we now describe the observed continuity between these populations in the Discussion section as follows:

Notably, the UMAP revealed a continuum between C23 and C18 within the intracardiac mesenchyme population. Given previous findings that the proximal coronary artery SMCs originate from preotic NCCs[8] and that pericytes give rise to coronary artery SMCs[23], this connection likely represent a differentiation trajectory from intracardiac mesenchyme to coronary artery SMCs via a pericyte-like intermediate stage.
(P9, L15–19)

The continuity between intracardiac mesenchyme and coronary artery SMCs through a pericyte-like intermediate state is consistent with previous developmental studies showing that proximal coronary artery SMCs originate from preotic NCCs and may arise through pericyte intermediates[8,21].
(P18, L24-27)

7. The authors nicely show downregulation of Hox gene expression in NCC cells entering the heart. Can they add discussion of any insights into this from prior studies of loss or gain of Hox gene function?

We have added the following discussion on the roles of anterior *Hox* genes in cardiovascular development, together with appropriate references on loss or gain of *Hox* gene function:

The regional identities of pharyngeal NCCs that contribute to cardiac development are established by Hox genes and their associated regulatory networks. Genetic studies have demonstrated essential roles for the anterior Hox genes in patterning the pharyngeal arch artery system and semilunar valve structures. Loss of Hoxa1 and Hoxb1 results in severe defects in pharyngeal arch artery development[42], whereas ectopic or sustained expression of Hoxb1 in NCCs disrupts cardiovascular morphogenesis and causes malformations of the great arteries and

semilunar valves[43]. Likewise, Hoxa3 contributes to proper patterning of the pharyngeal arch region and its NCC-derived derivatives[44,45]. These findings underscore the importance of precise spatial and temporal regulation of Hox genes during cardiovascular development. Among pharyngeal NCCs contributing to cardiac development, cushion-independent NCC derivatives (great artery SMCs and the aorticopulmonary septum) retain their origin-specific Hox-codes. In contrast, cushion-associated NCC derivatives (coronary artery SMCs and valvular/subvalvular interstitial cells) downregulate Hox expression and transition toward region-specific GRNs involving TFs such as Tbx20 and Gata4, whose expression is known to be induced by BMP signaling in cardiomyocytes[46,47]. Bmp2 and Bmp4 are expressed in the regions of the pericardial reflection traversed by NCCs en route to the cardiac cushion[48]. Together, these observations suggest that appropriate repression of Hox programs, coupled with activation of cardiac-specific regulatory networks, is required for normal differentiation of cushion-associated NCC derivatives.
(P18, L30 – P19, L16)

42. *Roux, M. et al. Hoxa1 and Hoxb1 are required for pharyngeal arch artery development. Mech. Dev. 143, 1–8 (2017).*
43. *Zaffran, S., Odelin, G., Stefanovic, S., Lescroart, F. & Etchevers, H. C. Ectopic expression of Hoxb1 induces cardiac and craniofacial malformations. genesis 56, (2018).*
44. *Chisaka, O. & Capecchi, M. R. Regionally restricted developmental defects resulting from targeted disruption of the mouse homeobox gene hox-1.5. Nature 350, 473–479 (1991).*
45. *Kameda, Y., Watari-Goshima, N., Nishimaki, T. & Chisaka, O. Disruption of the Hoxa3 homeobox gene results in anomalies of the carotid artery system and the arterial baroreceptors. Cell Tissue Res. 311, 343–352 (2003).*

In addition, we previously generated conditional *Hoxa2* overexpression mice and demonstrated that ectopic *Hoxa2* expression in *Hox*-negative PA1 cranial neural crest derivatives induced PA2-like structures, indicating a partial homeotic transformation (Kitazawa et al., Developmental Biology, 2015; 10.1016/j.ydbio.2015.04.007). Because cardiovascular phenotypes were not examined in that study, we have now resumed breeding of these mice for detailed cardiovascular phenotypic analyses.

In parallel, we have also established knockout mice for a downstream target of *Hoxa2*, which are expected to complement the *Hoxa2* gain-of-function model and provide further insight into the regulatory mechanisms underlying cardiac NCC differentiation and patterning. Although we

have obtained preliminary observations from these models, a comprehensive analysis is still ongoing, and we therefore prefer to reserve these results for a future study with more detailed investigation.

8. Figure 3Y could be simplified to more clearly distinguish the two types of Meis binding sites. For example, it may be helpful to reorder the mesenchymal cell types based on Hox expression.

To improve clarity and better distinguish the two types of Meis binding sites, we have reordered the heatmap of motif enrichment based on the hierarchical clustering with the updated JASPAR2024 database. In parallel, we have revised the heatmap of transcription factor gene expression to provide a more consistent and interpretable presentation. These diagrams were now presented as Figures 4k and 4l of the revised manuscript.

9. The authors provide nice in vitro and in vivo evidence for an upstream role of Meis transcription factors in regulating Sox9 expression. Can the authors identify from the enhancer sequence (or their transcriptomic dataset) any of the non-Hox transcription factors that Meis may be working with here? Please discuss the significance of Sox9 expression in epicardium driven by the same enhancer. Might this regulation also operate in second heart field progenitor cells where both genes are expressed? It is not evident in Figure 7 that Sox9-EGFP is also expressed in epicardium.

The distal *Sox9* enhancer containing Meis2 binding site that we identified (chr11-112850240-112851186) also contains several consensus motifs including predicted Hand2- and Nfatc1-binding sites. However, to our knowledge, these transcription factors have not been reported as non-Hox partners of Meis proteins. Their recruitment to this enhancer, as well as potential cooperative interactions with Meis transcription factors, were not examined in the present study and remain subjects for future investigation.

As the reviewer pointed out, Sox9 was expressed not only in intracardiac NCCs but also in the epicardium (revised Figure S13). Consistent with this expression pattern, distal *Sox9* enhancer was accessible in both intracardiac mesenchyme and epicardial cells (revised Figure S13). Especially, $Wt1^{low}$/$Sox9^{high}$ mesenchymal cells, likely derived from the epicardium via epithelial-mesenchymal transition, also exhibit chromatin accessibility at this enhancer comparable to that observed in NCC-derived mesenchymal cells. These findings suggest that the same regulatory

element may function across multiple cardiac lineages.

We have addressed these points in the revised manuscript as follows, including additional supporting data in the supplementary figures.

Enhancer activity in the epicardium corresponds to Sox9 expression and an open chromatin peak at the putative distal enhancer region in clusters 22 and 5 in Figure 1d, which represent $Wt1^{high}$ epicardial cells and intracardiac mesenchyme likely including $Wt1^{low}$ epicardial EMT derivatives, respectively (Figure S13).
(P16, L28–32)

10. Could this approach yield similar data for Osr1? Please clarify if there is any experimental evidence supporting the predicted negative regulation of Sox9 by Osr1 in the heart illustrated in Figure 8.

There are currently no experimental data demonstrating Sox9 repression by Osr1 in the heart. However, such an effect has been reported in tongue and limb mesenchyme (Liu et al., PNAS, 2013), as noted in P16, L5–7. Although direct experimental validation, such as Osr1 overexpression in cardiopharyngeal NCCs, would provide stronger evidence, preparation of this specific NCC lineage is difficult. Given this limitation, we instead performed *in silico* gene perturbation analysis using CellOracle, which predicted antagonistic roles for Sox9 and Osr1 during lineage bifurcation from pharyngeal NCCs. Because this regulatory relationship has not yet been experimentally validated in the cardiac context, we revised the illustration accordingly by adding a question mark to indicate the hypothetical nature of this antagonism (Figure 9 in the revised version).

11. Concerning the links between valve mesenchyme and skeletogenic programs it would be relevant to cite the earlier work of Lincoln and Yutzey (reviewed in PMID: 16643886):

We cited the suggested work in the relevant portion of the Discussion section as follows:

By analogy, $Sox9^{high}/Scx^{high}$ NCCs at the base of semilunar valves may form a structural attachment unit linking cushion tissues to valvular leaflets[57].
(P20, L30–32)

57.Lincoln, J., Lange, A. W. & Yutzey, K. E. Hearts and bones: Shared regulatory mechanisms in heart valve, cartilage, tendon, and bone development. Dev. Biol. 294, 292–302 (2006).

12. In order to increase accessibility of the dataset the authors are encouraged to include a browser link.

We agree with the reviewer that improving dataset accessibility is important for facilitating exploration of NCC diversity. Therefore, we have already uploaded our original fastq sequence files and count matrices in the DDBJ and GEO servers. In addition, we will upload our gene expression datasets projected onto the UMAP in UCSC cell browser, enabling readers to more easily visualize and interrogate the dataset.

Minor points:

13. The authors could rephrase the title since the term topographical genetic switch is unclear.

We thank the reviewer for this suggestion. To improve clarity, we revised the title as follows:

Hox–Meis-relayed spatial gene regulatory transition underlies cardiopharyngeal neural crest diversification revealed by multimodal analysis

14. In the introduction, with reference to the De Bono study, please note that Tbx1 was shown to regulate pharyngeal NCC differentiation stage transitions non-cell autonomously.

According to the reviewer's suggestion, we rephrased the relevant section of the introduction as follows:

De Bono et al. elaborated the transition of pharyngeal NCCs through multiple differentiating stages toward SMC fates, identifying Tbx2 and Tbx3 as key TFs in this process[13]. They also showed that Tbx1, the gene for 22q11.2 deletion syndrome, regulates pharyngeal NCC differentiation stage transitions non-cell autonomously[13].

(P5, L1–4)

Reviewer #1 (Significance (Required)):

Iwase et al have used multiomics and spatial transcriptomics to comprehensively map neural crest cell contributions to the mouse heart and great arteries. This careful and detailed analysis reveals changes in the transcriptional profile of neural crest cells as they give rise to different regions and cell types in the heart and great vessels. The study significantly builds on a number of recent scRNA-seq analyses of neural crest cell development and includes development of a new informatic tool for regulatory network investigation. Among the new findings documented are downregulation of Hox gene expression in intracardiac crest cells and regulation of Sox9 by Meis transcription factors.

Again, thank you for giving us the opportunity to strengthen our manuscript with your valuable comments and queries. We have worked hard to incorporate your feedback and hope that these revisions persuade and satisfy you.

To Reviewer #2

Reviewer #2 (Evidence, reproducibility and clarity (Required)):

Summary

In this manuscript, Iwase et al. cleverly make use of different modalities, spatial transcriptomics and single-cell omics datasets, in conjunction with a well-established Wnt1-Cre;R26R-EYFP line to trace neural crest cells (NCCs) contributing to the cardiovascular system during embryonic development in the mouse.

By doing so, the authors identified a bifurcation between cardiac NCCs contributing to the OFT cushions and forming the aorticopulmonary (AP) septation complex. Thus, the authors split "intracardiac NCCs" into two different NCC programs/compartments, even though both reside in the broad OFT region. The NCCs that enter and associate with the OFT cushions undergo a Hox off transition (Hox-positive to Hox-negative once intracardiac), with a corresponding shift in Meis binding and GRN wiring. The authors propose these cells pass through a Meis2-Sox9-Scx "skeletogenic progenitor-like" intermediate and contribute to semilunar valves and coronary artery smooth muscle. By contrast, the NCCs assigned to aorticopulmonary septum (APS) formation and great vessel smooth muscle retain a distinct Hox codes.

Thank you for your encouraging feedback and comments. We have responded to your comments below.

Major Comments

1. The manuscript would benefit from clearer delineation between the different NCC contributions, particularly for non-specialist readers. The distinction between (i) CNCCs in OFT cushions and (ii) CNCCs forming the aorticopulmonary (AP) septation complex is not adequately explained. While both populations contribute to OFT septation, according to the authors, they represent distinct compartments with different developmental trajectories. The authors could clarify this using anatomically labelled hearts at the stages they conduct their analysis, along with additional text and schematics explaining what is meant by each compartment. This would greatly enhance the accessibility of the manuscript.

To clarify the distinction between the two components of intracardiac NCCs, cushion-associated

mesenchyme and cushion-independent aorticopulmonary (AP) septum, we substantially revised the description of cluster characterization (P9, L24–P10, L6). We added new figures (Figure S5) showing their spatial relationships and distinct gene expression signatures, including E12.5 Xenium data demonstrating *Vegfc* expression in cushion-associated NCCs and *Tcf24* expression in AP septum NCCs.

In addition, we revised the schematic diagram of intracardiac NCC distribution in Figure 4m and added Figure S9, which spatially delineates three major NCC trajectories with distinct Hox codes: (1) migration of preotic NCCs into the outflow tract cushions, (2) migration of postotic NCCs into the outflow tract cushions, and (3) protrusion of the AP septum from the dorsal wall of the aortic sac. We also incorporated additional explanatory text and cited relevant review articles on cardiac outflow tract development. We hope that these revisions substantially improve the clarity and accessibility of the manuscript, particularly for non-specialist readers.

2. The Methods state that "Decomposition of cell clusters of scRNA-seq was performed by RCTD to map them onto the Xenium dataset," but this description is insufficient. The authors should clarify whether RCTD was applied separately for each developmental stage (i.e., E11.5 scRNA-seq reference for E11.5 Xenium, E12.5 reference for E12.5 Xenium), or whether a pooled reference was used across stages. This clarification is important because RCTD performance depends critically on the correspondence between reference and target datasets. Using a multi-stage integrated reference to deconvolve stage-specific spatial data could introduce artifacts, as cell states and cluster compositions vary considerably across developmental timepoints.

The pooled scRNA-seq data of NCCs or non-NCCs at E11.5 and E12.5 were used for RCTD to decompose for the spatial allocation in the Xenium dataset. We agree with the reviewer that a multi-stage integrated reference may introduce artifacts. In fact, datasets at E11.5 and E12.5 were similarly distributed in UMAP space and exhibited similar transcriptomic signatures, whereas those at E14.5 and E17.5 demonstrated different characteristics in the integrated UMAP in Figure. 1c. Therefore, we used only E11.5 and E12.5 datasets for RCTD decomposition. We added the bellow sentence in Method section.

The pooled scRNA-seq data of NCCs or non-NCCs at E11.5 and E12.5 were used for RCTD.
(P33, L6)

3. Figure 2j-m needs annotations and schematics. It is currently very difficult to identify the different compartments. See Figure C in Chen et al. for an example of this approach. In addition, what are the fine clusters from 1 to 20? Which ones are NCC-derived?

We added anatomical annotations to the revised Figure 2a, b, j–m to facilitate identification of the different compartments. The fine clusters labeled 0–20 in the previous Figure 2j–m corresponded to the multiome clusters (NCC and non-NCC) shown in Figure 1d. In the revised manuscript, the same dataset was re-clustered into 23 clusters (0–22), which were subsequently used for decomposition analysis to predict cell compartments with maximum likelihood. We also revised the color scheme of the segmented cells in Figure 2j–m to improve visual distinction between compartments and facilitate interpretation of the spatial distribution patterns.

Putative NCCs were identified through the following procedure:

1. EYFP expression was estimated across 39 cell types in the Xenium dataset by integration with the single-cell multiome dataset (including both NCCs and non-NCCs) using Tangram.
2. Xenium clusters enriched for EYFP expression, defined as clusters whose mean estimated EYFP level exceeded the threshold corresponding to the 65th percentile across all spots, and consistent with known neural crest derivatives were extracted as putative NCC populations.
3. For each spot within these EYFP-enriched Xenium clusters, RCTD was used to estimate the corresponding multiome cluster identity.

Thus, the diagrams in Figure 2j–m indicate the most likely multiome subpopulation assignment for each putative NCC spot, rather than categorizing the multiome clusters themselves as NCC- or non-NCC-derived. This description has been included in the Method section (P33, L6–14).

4. The panels show pharyngeal markers, OFT/intracardiac markers, and SMC markers in Figures 2n-y, but could the authors show the proportion of NCC-derived (YFP+) cells for each cluster? Could the authors also map only the YFP+ cells on the Xenium data? It would be useful to see the proportion of YFP-positive (NCC-derived) cells for each delineated compartment. YFP-positive cells appear to exist at the boundary between LV and RV in the septum, this observation would benefit from proper quantification.

Our Xenium analysis could not detect EYFP signals; therefore, as noted above, we estimated EYFP expression by integrating the scRNA-seq and Xenium datasets using Tangram. To clarify the relative enrichment of distribution for each Xenium cluster, we summarized the mean estimated EYFP expression as bar plots (Revised Figure S2q, r). Consistent with their established neural crest origin, Xenium clusters annotated as neuron (38), ganglion (32), and Schwann cell (17) showed high estimated EYFP expression. In addition, Xenium clusters 15 (SMC) and 25 (cushion mesenchyme) were also enriched for EYFP expression.

Unfortunately, currently available algorithms for Xenium data analysis do not reliably allow visualization or extraction of gene expression profiles exclusively from putative EYFP-positive cells. Instead, we improved the visualization of the spatial distribution of putative EYFP-positive cells by replacing centroid-based signal display with segmentation-based rendering (Revised Figure 2f, g).

As pointed out by the reviewer, putative YFP-positive cells appear to be present at the boundary between the LV and RV within the interventricular septum. However, the estimated EYFP signals in this region were substantially lower than those observed in well-established neural crest derivatives, suggesting that most of these signals likely represent background noise, although a minor population of sparsely distributed neural crest-derived cells cannot be excluded. More precise characterization of potential neural crest derivatives in the ventricular region will require future investigation.

5. Figure 3 is confusing because it integrates data from multiple overlapping stages (E8.5 to P7). While the authors identify distinct compartments, pharyngeal mesenchyme, intracardiac mesenchyme, and SMCs, it is unclear why stages beyond E14.5 and E17.5 (corresponding to the initial single-cell omics and Visium analysis) were included. Although leveraging additional datasets is a clever approach, the integration of data from such disparate developmental timepoints confounds interpretation. For example, Cluster 6 appears to include cells from both P7 and E12.5 stages. Given that the Visium data represent hearts at E14.5 and E17.5, it is problematic to map clusters derived from other stages onto these spatial datasets.

We integrate data from multiple overlapping stages (E8.5 to P7) in Figure 3 to capture diachronic cell identity and also stage-specific features, particularly within intracardiac mesenchyme and SMC populations. This approach enabled us to assess core lineage relationships, including trajectories linking pharyngeal mesenchyme to intracardiac mesenchyme and SMCs, each

comprising multiple distinct subpopulations. These findings support the biological relevance of the integration as a framework for understanding lineage relationships across developmental time. However, we agree with the reviewer that it is problematic to map clusters derived from other stages onto these spatial datasets. Indeed, we restricted the spatial mapping analysis to cells from E14.5 and E17.5 within the integrated dataset, thereby ensuring consistency with the developmental stages represented in the Visium data. To address concerns about this issue, we have clarified this point in the Methods section by adding the underlined words in the following sentence.

Spatial mapping of scRNA-seq data onto Visium sections was performed using <u>only E14.5 and E17.5 datasets and</u> the RCTD algorithm, as described above.
(P 34, L12–13)

6. Throughout the manuscript, the authors describe "lineage relationships" between cell populations, but these are in fact developmental trajectories inferred computationally (via UMAP connectivity and RNA velocity), not true lineage relationships. This distinction is critical and should be explicitly stated.

We agree with the reviewer that the present “lineage relationships” described in the original manuscript were primarily inferred from computational analyses, including UMAP connectivity and RNA velocity, rather than being directly demonstrated by lineage-tracing experiments. We also acknowledge that some RNA velocity results were not fully consistent with known in vivo developmental trajectories.

We strengthened the biological validation of the inferred trajectories by incorporating extensive spatial verification of gene expression using immunohistochemistry and Xenium in situ hybridization analyses to confirm the identity and localization of each cell population. We also refined the computational analyses to better resolve regional differences in NCC dynamics and added comprehensive schematic illustrations based on established models of heart development from previous studies, with appropriate citations throughout the manuscript.

In the revised manuscript, we have added much spatial verification of gene expression by immunohistochemistry and Xenium in situ hybridization data to confirm the identity of each cell population, and also include proper citation in the appropriate context. We also improved computational analysis to clarify the regional difference in NCC dynamics with comprehensive

schematic illustration based on heart developmental processes established by previous literatures. In response to the reviewer's comment and these revisions, we have carefully rephrased the relevant descriptions to clarify that the observed relationships represent computationally inferred developmental trajectories rather than definitive lineage relationships as follows.

(original) *RNA velocity analysis in conjunction with developmental context, revealed lineage relationships among these groups (Figure 3c, d).*
(revised) *RNA velocity analysis in conjunction with developmental context, <u>inferred global</u> lineage relationships among these groups (Figure 3c, d), <u>consistent with developmental trajectories in vivo.</u>*
(P8, L21–23)

(original) *..., we present a comprehensive map of cardiopharyngeal NCC <u>lineages</u> ...*
(revised) *..., we present a comprehensive map of cardiopharyngeal NCC <u>populations</u> ...*
(P18, L1)

(original) *Overall, this study proposes a new framework for understanding cardiac NCCs heterogeneity based on the association with the cardiac cushion and the accompanying transition in Hox gene expression and regulatory programs. Our findings provide a basis for systematically dissecting the developmental diversity of cardiac NCCs.*
(revised) *Overall, this study proposes a new framework for understanding cardiac NCCs heterogeneity based on <u>developmental route, Hox-code retention, and region-specific</u> regulatory programs. <u>Importantly, the developmental relationships and differentiation pathways described here are inferred from integrated computational analyses, including transcriptomic similarity, UMAP connectivity, and RNA velocity, rather than direct lineage-tracing experiments. Within this framework, our findings suggest distinct differentiation trajectories leading to great artery, ductus arteriosus, and coronary artery SMCs, as well as the aorticopulmonary septum and valvular/subvalvular mesenchyme.</u>*
(P20, L33 – P21, L7)

We also rephrased additional relevant sections throughout the manuscript in accordance with the reviewer's comment (Please see below).

For example, the statement "we propose that intracardiac NCCs within C10 and C22 differentiate via C2 into valvular (C21) and subvalvular (C18) interstitial cells" should be framed as a computational inference, not an established lineage relationship. Without clonal lineage tracing data, these claims cannot be verified.

We have changed the word “propose” to “infer” (P10, L24).

Similarly, the claim that "NCCs contributing to the AP septum are distinct from other intracardiac NCCs in that they do not populate the cardiac cushions but remain continuous with NCCs populating the distal outflow tract cushion" lacks direct lineage evidence. What experimental data support this assertion?

We agree with the reviewer that the original statement lacked direct lineage evidence, although the anatomical distinction between the outflow tract cushions and the AP septum—a protruding structure arising from the dorsal wall of the aortic sac—is well established. In the revised manuscript, we therefore removed this speculative statement and rewrote the section to more accurately describe the developmental process with appropriate references. The spatial and temporal features of AP septum formation are now also illustrated in Figure S9a (UMAP plots and schematic illustration) and described as follows:

From E12.5 to E14.5, postotic cells showed a progressive emergence of the aorticopulmonary septum–associated cluster C16 from transitional states.
(P11, L32 – P12, L1)

In addition, we further validated the identity of the C16 cluster as AP septum–associated NCCs by incorporating additional marker analyses together with spatial verification using Xenium in situ analysis. In addition to *Penk* and *Sfrp2*, which were previously reported by Chen et al., C16 also highly expressed *Postn*, similar to other intracardiac clusters. Furthermore, C16 was characterized by relatively high expression of *Tcf24* and low expression of *Vegfc*. Based on the reviewer’s comment and these additional experimental data, we revised the relevant Results section as follows:

(original) *Within the intracardiac mesenchyme group, C16 exhibited high expression of Penk and*

Sfrp2 (Figure S3i and Table S6), corresponding to the cluster annotated as the aorticopulmonary septum in the previous study by Chen et al[14]. This annotation was further supported by enriched expression of Hox4 and Hox5 paralogs, consistent with its origin between PA4 and PA6 (Figure 3t, u). The aorticopulmonary septum originates as a protrusion from the dorsal wall of the aortic sac and is primarily derived from NCCs[6,18–20]. This septal structure fuses with the distal outflow tract cushions to divide the common arterial trunk into the aortic and pulmonary channels. Notably, NCCs contributing to this septum are distinct from other intracardiac NCCs in that it does not populate the cardiac cushions but remain continuous with NCCs populating the distal outflow tract cushion, suggesting that C16 represents this distinct NCC-derived population.

(revised) *C16 <u>was distinguished by</u> high expression of Penk and Sfrp2 (Figure S3i and Table S6), corresponding to the cluster annotated as the aorticopulmonary septum in the previous study by Chen et al[14]. <u>This cluster also exhibited robust expression of mesenchymal markers, including Postn, similar to other intracardiac clusters (Figure S3i). In addition, C16 showed relatively high expression of Tcf24 and low expression of Vegfc compared with the other intracardiac clusters (Figure S5a-e). These gene expression features of the aorticopulmonary septum were further validated by Xenium in situ hybridization (Figure S5f-j).</u>*

<u>Unlike other intracardiac NCCs that populate the distal outflow tract cushions,</u> the aorticopulmonary septum originates as a protrusion from the dorsal wall of the aortic sac and is primarily derived from NCCs<u> residing in PA4 and PA6[6,22–24]</u>. <u>This septal structure subsequently fuses with the distal outflow tract cushions to partition the common arterial trunk into the aortic and pulmonary channels. Consistent with this developmental origin, C16 was enriched for the expression of Hox4 and Hox5 paralogs (Figure 3t, u), indicating that NCCs in this population retain their Hox code, in contrast to other intracardiac NCCs, in which most Hox genes were downregulated (see later details).</u>

(P9, L24 – P10, L6)

In addition, the authors state that what De Bono et al. identified as "outflow smooth muscle" corresponds in their dataset to early intracardiac mesenchymal clusters C2, C10, and C22, present as early as E10.5, when mesenchymal NCC derivatives express immature SMC markers, and that these cells later differentiate into coronary artery SMCs around E14.5 (C23) as well as other non-muscle components. This claim is not verified, whether these cells are indeed the ones differentiating into coronary artery SMCs is based solely on computational inference from C2,

C10, C22 to C23.

To validate that C23 corresponds to coronary artery SMCs, we performed additional immunostaining analyses for Myh11, Sost, and Reln, which distinguish great artery SMCs, ductus arteriosus SMCs, and coronary artery SMCs, respectively, consistent with their transcriptomic identities. Regarding differentiation of NCC-derived mesenchymal cells into coronary artery SMCs, we previously demonstrated using chick–quail chimera experiments and specific Cre-reporter mouse lines that proximal coronary artery SMCs originate from preotic NCCs rather than postotic NCCs (Arima Y et al. *Nature Communications* 3:1267, 2012). In addition, coronary artery SMCs have been reported to differentiate through pericyte intermediates (Volz KS et al., *eLife* 4:1–22, 2015). Consistent with these findings, our present lineage-tracing analyses using *Sox9-CreERT2; Ai14* and *Scx-CreERT2; Ai14* mice demonstrated that progenies of $Sox9^{high}$ and Scx^{high} intracardiac cushion mesenchymal cells contributed to the coronary artery SMCs as well as the surrounding mesenchyme.

We agree with the reviewer that the relationship between the early intracardiac mesenchymal clusters (C2, C10, and C22) and C23 is primarily inferred from computational trajectory analyses and is not demonstrated by direct clonal lineage tracing. Accordingly, we revised the Discussion to avoid overstatement and to clarify that these lineage relationships are inferred based on computational analyses together with prior experimental findings and the additional validation data described above. The revised text is as follows:

(original) *Our integrated map incorporates previously published lineage analyses of cardiac NCCs at early and late stages[13,14], providing continuity through complementary single-cell and spatial transcriptomic data, although our interpretation of certain clusters differs from those of prior studies. For example, the cell population identified by De Bono et al. as outflow smooth muscle[13] corresponds in our dataset to early intracardiac mesenchymal clusters C2, C10, and C22, present as early as E10.5, when mesenchymal NCC derivatives express immature SMC markers. These cells later differentiate into coronary artery SMCs around E14.5 (C23) as well as other non-muscle components. Despite such differences in interpretation, the integrated map robustly captures lineage relationships, supported by accumulated developmental and anatomical evidence regarding cardiac outflow tract formation, particularly in relation to the outflow tract cushion.*

(revised) *Our integrated map incorporates previously published lineage analyses of cardiac NCCs at early and late stages[13,14], providing continuity through complementary single-cell and spatial transcriptomic data. The present study further extends these datasets by resolving the heterogeneity of intracardiac mesenchymal populations and their lineage relationships. For example, the cell population identified by De Bono et al. as outflow smooth muscle[13] corresponds in our dataset to early intracardiac mesenchymal clusters expressing immature SMC markers, which subsequently diverged into multiple derivatives including coronary artery SMCs. In addition, we identified distinct SMC populations corresponding to great artery SMCs, ductus arteriosus SMCs, and coronary artery SMCs, each characterized by unique molecular signatures such as Sost, Tfap2b/Ptger4, and Reln/Gja4, respectively. The continuity between intracardiac mesenchyme and coronary artery SMCs through a pericyte-like intermediate state is consistent with previous developmental studies showing that proximal coronary artery SMCs originate from preotic NCCs and may arise through pericyte intermediates[8,21]. Together, these findings provide a refined framework for understanding the diversification of cardiac NCC derivatives during outflow tract remodeling.*

(P18, L14–29)

7. Claims regarding marker expression in specific compartments (for example Hapln1 and Postn in cushions) require additional spatial validation at higher resolution than what is currently provided by the Visium data. Moreover, it is unclear whether these data are single-cell resolution; the authors need to clarify this. HCR staining would be ideal to confirm these expression patterns. Currently, all conclusions are based solely on gene expression without orthogonal spatial confirmation. At minimum, the authors should provide references from the literature supporting these expression patterns.

As reviewer suggested, the confirmation of spatial context for the gene expression patterns of scRNA-seqs data is important to validate. We further investigated the spatial expression patterns through Xenium in situ hybridization system. Among cardiac mesenchyme subpopulation, Postn was dominantly expressed, however, Tcf24 was specifically expressed in AP septum (C16) not in cushion (C2, 10, 18, 21 and 22) in scRNA-seq data. On the other hand, Vegfc was expressed except in the AP septum. To confirm these opposing expression patterns, we newly added Figure S5, showing Tcf24 and Vegfc expression revealed by Xenium.

According to the reviewer's suggestion, we added the sentence in the revised manuscript as follows:

C16 was distinguished by high expression of Penk and Sfrp2 (Figure S3i and Table S6), corresponding to the cluster annotated as the aorticopulmonary septum in the previous study by Chen et al[14]. *This cluster also exhibited robust expression of mesenchymal markers, including Postn, similar to other intracardiac clusters (Figure S3i). In addition, C16 showed relatively high expression of Tcf24 and low expression of Vegfc compared with the other intracardiac clusters (Figure S5a-e). These gene expression features of the aorticopulmonary septum were further validated by Xenium in situ hybridization (Figure S5f-j).*

(P9, L24–31)

8. Could the authors confirm the absence of the Sox9high/Scxhigh population in AP septum descendants?

Sox9^{high}/*Scx*^{high} NCCs are enriched not only in intracardiac NCC clusters C2, C10, and C14, but also in the AP septum-associated cluster C16, as stated in the manuscript as follows:

In the integrated UMAP, Sox9^{high}*/Scx*^{high} *NCCs were enriched in C2, C10, C14 and C16.*

(P17, L7–8)

However, based on our previous finding that proximal coronary artery SMCs originate from preotic rather than postotic NCCs (Arima Y et al. *Nature Communications* 3:1267, 2012), we infer that the intermediate population contributing to coronary artery SMCs is more likely derived from intracardiac NCC clusters C2 and C10 than from the pharyngeal arch 4/6-derived AP septum-associated cluster C16. To clarify this interpretation, we have added the following statement to the final paragraph of the Results section:

Together with our previous report that proximal coronary artery SMCs originate from preotic rather than postotic NCCs[8]*, these results suggest that the intermediate population contributing to coronary artery SMCs likely represents a subset of Hox-downregulated intracardiac NCCs corresponding to clusters C2 and C10.*

(P17, L29–33)

Minor Comments

Could the authors better justify their choice of stages (E11.5 to E17.5) for the single-cell multiomic assay? Given that OFT cushions are already populated by NCCs by E10.5 and that AP septum formation is already underway at this stage (see Development (2007) 134(8): 1593-1604), the rationale for beginning at E11.5 should be explicitly stated.

We agree that NCCs have already populated the OFT cushions and that AP septum formation is underway by E10.5. Our selection of stages from E11.5 to E17.5 was intended primarily to enable synchronous comparisons between pharyngeal and intracardiac NCC populations across developmental stages, rather than to capture the earliest spatiotemporal events of cardiopharyngeal NCC lineage establishment. We have clarified this rationale in the revised manuscript by revising the statement as follows:

(original) To elucidate the spatiotemporal dynamics of cardiopharyngeal NCC lineages, we performed single-cell multiome analysis on pharyngeal and cardiac tissues from E11.5 and E12.5 and ...

(revised) To <u>compare pharyngeal and intracardiac NCCs across developmental stages and characterize their temporal changes</u>, we performed single-cell multiome analysis on pharyngeal and cardiac tissues from E11.5 and E12.5 and ...
(P6, L3-5)

Spelling errors in Figure 2e: "ambious" should be "ambiguous"; "atrium venticle" should be "atrium/ventricle"; "ventricle" is misspelled in several locations.
The clustering resolution is very high, yielding many clusters that are difficult to distinguish based on the colour code alone. What does "ventricle_CL" refer to? What is the "marginal layer"? A clearer legend or supplementary table defining each cluster would be helpful.

To improve the clarity of the high-resolution clustering, we added Xenium cluster numbers to Figures 2c and 2d, enabling clusters to be distinguished not only by color but also by their anatomical locations. We also revised the cluster annotations in Figure 2e and corrected all

spelling errors, including “ambiguous,”. In addition, we replaced “ventricle_CL” with “Cardiac muscle 1” and the formerly misspelled “atrium ventricle CM” with “Cardiac muscle 2.” The annotation “marginal layer” was also revised to “Spinal cord, marginal layer” for clarity.

Figure 2j: The image is very dim.

We have improved the image quality and added regional annotations to enhance visibility in the revised figure.

Figure 3b: What do the numbers correspond to? Additionally, "mesenchyme" is misspelled.

We have revised Figure 3b to clarify different characteristics of mesenchymal subpopulations. We have also corrected the misspelling of “mesenchyme.”

Referees cross-commenting

I also agree with the other reviewers' comments. Many thanks.

Reviewer #2 (Significance (Required)):

The overall approach is sound and the datasets generated are valuable resources for the field. The manuscript presents interesting findings regarding NCC heterogeneity in cardiac development.

As I understand the authors' model:
OFT cushion-associated NCCs enter and associate with the OFT cushions, undergoing a Hox-positive to Hox-negative transition, accompanied by a shift in Meis binding and GRN rewiring. These cells pass through a Meis2-Sox9-Scx "skeletogenic progenitor-like" intermediate state and contribute to semilunar valves and coronary artery smooth muscle.
Aorticopulmonary septum NCCs, by contrast, retain distinct Hox codes (Hox4/5 enrichment) rather than becoming Hox-negative, and contribute to the AP septum and great vessel smooth muscle.

Strengths: The integration of multiple omics modalities with lineage tracing is a powerful approach, and the identification of a Hox-dependent bifurcation in NCC fate is a novel conceptual advance.

Limitations: The reliance on computational trajectory inference without orthogonal lineage validation, combined with the integration of datasets spanning very different developmental stages, limits the strength of some conclusions. The analysis also required more precise anatomical annotations to facilitate accessibility to the readers - to visualise better the distinguishable contribution of the cardiac NCCs to the OFT.

Advance
The study extends knowledge in the field by providing novel mechanistic insight into neural crest diversification in the context of cardiovascular development. The nature of the advance is primarily mechanistic, identifying a Hox-Meis regulatory switch that distinguishes cushion-associated from cushion-independent NCC lineages.

Audience
This work will be of interest to a specialised audience interested in neural crest cells and developmental biologists using omics approaches to address cell fate diversification in complex tissues.

Reviewer Expertise
Developmental biology, lineage analysis, mouse genetics. I do not have the expertise to assess the computational methods used in this paper.

Again, thank you for giving us the opportunity to strengthen our manuscript with your valuable comments and queries. We have worked hard to incorporate your feedback and hope that these revisions persuade and satisfy you.

To Reviewer #3

Reviewer #3 (Evidence, reproducibility and clarity (Required)):

Summary:

Iwase et al. presents a comprehensive multiomics analyses of cardiac neural crest cell (NCC) differentiation during cardiopharyngeal development. Using Wnt1Cre;R26R-EYFP mice, the authors isolated NCCs and non-NCCs at E11.5, E12.5, E14.5 and E17.5 stages and performed single-cell RNA-seq, ATAC-seq, spatial transcriptomics analyses. Spatial resolution of NCC-derived populations was achieved using Xenium (E11.5-E12.5) and Visium (E14.5-E17.5) platforms. Integration of single cell and spatial datasets identified distinct NCC-derived populations with defined spatial organization within the pharyngeal and intracardiac regions. The study concludes that Hox gene patterning underlies NCC subpopulation identity during cardiopharyngeal development and reveals a developmental transition from Hox-dependent to Hox-independent transcriptional regulation. Furthermore, the authors identify a Meis2-Sox9-Scx gene regulatory network making a skeletogenic progenitor-like intermediate that contribute to coronary smooth muscle and semilunar valve formation.

While the dataset is comprehensive and technically strong, several key conclusions are not always convincingly supported by enough data. As a result, some claims appear speculative and would benefit from additional experimental validation to strengthen the proposed developmental models.

I would strongly encourage authors to consider the following points to provide additional details that will strengthen their study:

Thank you for your encouraging feedback and comments. We have responded to your comments below.

Major comments:

- The authors should provide detailed FACS gating strategies and sorting conditions used to selectively isolate EYP-positive and EYP-negative NCC populations, including representative

plots and information on exclusion criteria (e.g., doublets, dead cells).

We added the sorting gates to the revised Figure S1 and described the detailed FACS gating strategy and sorting conditions in the revised Methods section as follows:

(original) EYFP-positive and -negative single cells were sorted using a FACSAria II or FACSMelody (BD Biosciences), freshly processed or cryopreserved.

(revised) Single-cell suspensions were stained with 7-AAD (BD Pharmingen) for 3 min at 4℃, and EYFP-positive and -negative single cells were sorted using a FACSAria II or FACSMelody (BD Biosciences). The sorting strategy was as follows:
Step 1. All events were gated by forward scatter (FSC) and side scatter (SSC) including area (A), height (H), and width (W) to obtain FSC singlets and remove doublets.
Step 2. FSC singlets were gated for the 7-AAD negative fraction to isolate viable cells.
Step 3. Viable cells were gated to isolate EYFP-positive NCCs or EYFP-negative non-NCCs.
Sorted cells were freshly processed or cryopreserved for the following procedure.
(P28, L21–30)

- Although the authors isolated nuclei for both scRNA-seq and ATAC-seq, the number of cardiomyocytes within the EYFP-negative population is unexpectedly low. The authors should clarify potential technical or biological reasons for this underrepresentation (e.g., nuclei isolation efficiency, sorting strategy, filtering criteria, or developmental stage-specific composition).

The unexpectedly low proportion of cardiomyocytes within the EYFP-negative population likely reflects the restricted sampling region used in this study. Specifically, we dissected cardiopharyngeal tissue and the outflow tract region rather than the whole heart as shown in Figure 1a, which likely introduced a sampling bias that reduced the representation of cardiomyocytes in the dataset.
Although the FACS gating strategy could potentially influence the recovery of specific cell types, we consider this possibility unlikely because cardiomyocyte populations were successfully detected in our dataset. In addition, during the quality-control process for scRNA-seq data, we applied a mitochondrial gene threshold of 25% to exclude low-quality cells. While mature cardiomyocytes typically exhibit high mitochondrial gene expression, embryonic cardiomyocytes

at the analyzed developmental stages are immature and therefore were unlikely to be disproportionately excluded by this criterion. Consistent with this interpretation, distinct cardiomyocyte clusters remained detectable after filtering, indicating that cardiomyocytes were retained through the quality-control process.

Based on these considerations, we conclude that the low abundance of EYFP-negative cardiomyocytes primarily reflects the limited anatomical region sampled. We have clarified this point in the revised Results section as follows:

The overall proportion of cardiomyocytes was low, likely reflecting the restricted sampling of the cardiac outflow tract region (Figure 1a).

(P6, L23–25)

- In figure 1, the authors present results from unsupervised clustering of 9,420 cells into 21 distinct clusters, many of which are broadly labeled as "mesenchymal cells". The authors should refine this nomenclature by providing more specific annotations or defining criteria, as this broad classification limits interpretability of the identified subpopulations.

In the revised manuscript, we further refined the clustering analysis by subdividing the original clusters C5 and C13 into two subclusters each, resulting in a total of 23 clusters in the UMAP shown in Figure 1d. This refinement improved the resolution and interpretability of the identified cell populations. In addition, we replaced the broad "mesenchymal cells" annotation with more specific classifications, including pharyngeal mesenchymal cells, intracardiac mesenchymal cells, and smooth muscle–like cells. To further clarify lineage relationships, we also provided UMAPs separately displaying NCC and non-NCC populations in Figure 1e, as suggested by the reviewer.

- To integrate spatial annotated Xenium datasets with scRNA-seq data, the authors used Tangram, enabling estimation of the spatial distribution of EYFP-positive NCCs within the pharyngeal region of E11.5 and 12.5 embryos. However, the E11.5 section show a relatively low number of EYFP-positive cells (Figure 2f). The authors should clarify whether this reflects technical limitations (e.g., probe design, segmentation efficiency, or integration parameters) or biological factors and explain how this affects interpretation of the spatial analyses.

- The author used RCTD tool to decompose the scRNA-seq dataset into NCC and non-NCC components and mapped these onto the Xenium dataset. However, panels j and l in Figure 2 show

low signal in the E11.5 sections. The authors should clarify whether this reflects technical limitations of the RCTD deconvolution, differences in sampling, or biological factors, and discuss how this result impacts of interpretation of the spatial mapping results at this stage.

In the original manuscript, estimated EYFP expression levels were visualized using a minimum cutoff of 0.1, with the remaining values mapped onto a 100-step color scale. However, this approach resulted in apparently weak signal intensity in the E11.5 sections because high-level noisy signals, including signals detected in the atrial lumen and outside the embryo, broadened the dynamic range of the visualization. In the revised manuscript, we applied an upper cutoff at the 90th percentile to reduce the influence of these noisy signals, resulting in improved visualization of EYFP-positive regions in Figure 2f, with signal intensity now comparable to that observed in Figure 2g. In addition, we further improved the spatial visualization of putative EYFP-positive cells by replacing centroid-based signal display with segmentation-based rendering in the revised Figure 2f, g. Importantly, these revisions affected only the visualization method and did not alter the underlying analyses or conclusions, as the integration and downstream analyses were performed using the original quantitative data.

We also clarified the procedure used for NCC estimation and spatial mapping in the Methods section as follows:

Putative NCCs were identified through the following procedure:
Step 1. EYFP expression was estimated across 39 cell types in the Xenium dataset by integration with the single-cell multiome dataset (including both NCCs and non-NCCs) using Tangram.
Step 2. Xenium clusters enriched for EYFP expression, defined as clusters whose mean estimated EYFP level exceeded the threshold corresponding to the 65th percentile across all spots, and consistent with known neural crest derivatives were extracted as putative NCC populations.
Step 3. For each spot within these EYFP-enriched Xenium clusters, RCTD was used to estimate the corresponding multiome cluster identity.
(P33, L7–16).

- The authors integrated their data with publicly available scRNA-seq datasets of NCCs from E8.5 to P7 hearts and present results from unsupervised clustering of 67,208 cells into 28 distinct clusters. Figures 3a and 3b show that cardiomyocyte (C26) is included in NCC-derivatives. The authors should clarify whether this reflects technical issue when they made FACS.

In our original datasets in Figure 1d, only a small number of NCCs were detected within the cardiomyocyte cluster (corresponding to C26 in Figure 3b), which was otherwise predominantly composed of non-NCCs. Cardiomyocytes assigned to C26 were also present in both publicly available scRNA-seq datasets included in the integrated analysis. Previous studies have reported that a limited subset of NCCs can differentiate into cardiomyocytes (Tomita, Y. et al., J. Cell Biol. 170:1135–1146, 2005; Tamura, Y. et al., Arterioscler. Thromb. Vasc. Biol. 31:582–589, 2011). Therefore, we consider that C26 likely represents a small population of NCC-derived cardiomyocytes rather than contamination caused by technical issues during FACS isolation, although the low cell number precluded further characterization.

To clarify this point, we added the following statement to the Results section:

Only a few NCCs were detected within cardiomyocyte clusters, which were predominantly composed of non-NCCs, consistent with previous reports demonstrating NCC differentiation into cardiomyocytes[15,16].

(P6, L20–23)

- The authors used RNA-velocity to infer relationship among the identified clusters. However, this analysis requires particular caution given that data were generated from multiple datasets obtained under different conditions. Several conclusions drawn from the RNA-velocity analysis are not convincing, as illustrated in Figures 3c and 3d, where the inferred velocity directions appear inconsistent with the proposed developmental model (e.g., trajectory from cluster 23 toward 18 or from 4 toward 6). The authors should clarify these discrepancies, justify the integration of heterogenous datasets and reassess the interpretation of the inferred lineage relationships.

In the default setting, the integration workflow provided by Seurat which is widely used for scRNA-seq analysis employs canonical correlation analysis (CCA). CCA effectively corrects batch effects across datasets generated from different experimental platforms. However, it sometimes causes overcorrection to attempt to forcibly integrate different cell populations that are not shared among datasets (Andreatta, Bioinformatics, 2021). To minimize overcorrection for multiple datasets obtained under different experimental conditions, we applied reciprocal principal component analysis (RPCA) method recommended for comparative integration of

heterogeneous scRNA-seq datasets (Luecken et al., Nature Methods, 2021). This selection is suitable for the integration of multiple datasets provided by different independent studies as in case of the present study.

To infer relationship among the identified clusters, we then used RNA velocity analysis of scRNA-seq data fundamentally based on splicing dynamics. The original framework assumes that transcriptional induction and repression persist long enough for cells to reach active (transcribing) or inactive (silenced) steady-state equilibrium. However, this assumption is often violated during cell differentiation, where transient cell populations frequently exhibit rapidly changing mRNA levels that do not reach steady-state equilibrium. To address this limitation, the scVelo method was developed (Bergen et al., 2020, *Nature Biotechnology*), and we applied this approach to the integrated NCC datasets in the present study. This analysis successfully inferred directional flows from the pharyngeal mesenchyme toward SMCs and intracardiac mesenchyme through transitional states (Figure 3c,d). However, as the reviewer correctly pointed out, the analysis predicted a directional flow from C23 to C18, apparently opposite to the biological directionality supported by previous findings (see P9, L15–19).

We consider this discrepancy to reflect intrinsic limitations of RNA velocity analysis. As discussed by Bergen et al., in systems containing multiple lineages and cellular processes, differences in gene regulatory networks among heterogeneous cell states can generate multiple trajectories in phase space owing to distinct splicing kinetics. In addition, incompletely captured splicing kinetics may represent only a limited portion of the overall dynamics, particularly near terminal differentiation states. In such cases, phase portraits of unspliced versus spliced transcripts may appear nearly linear rather than curved, potentially leading to erroneous assignment of positive or negative RNA velocities. Consistent with this limitation, we obtained opposite directionalities between C18 and C23 depending on whether steady-state or dynamical models were applied and according to different parameter settings. Through these repeated computational re-evaluations of lineage directionality, we concluded that RNA velocity analysis is suitable for capturing the global landscape of differentiation flow, but that accurate inference of local lineage directionality may require careful model selection and parameter optimization to ensure consistency with established biological evidence.

To avoid arbitrariness and potential confusion, we removed the arrow between C18 and C23 from the revised Figure 3d. Instead, we now describe the observed continuity between these populations in the Results and Discussion sections as follows:

Notably, the UMAP revealed a continuum between C23 and C18 within the intracardiac mesenchyme population. Given previous findings that the proximal coronary artery SMCs originate from preotic NCCs[8] *and that pericytes give rise to coronary artery SMCs*[23]*, this connection likely represent a differentiation trajectory from intracardiac mesenchyme to coronary artery SMCs via a pericyte-like intermediate stage.*

(P9, L15–19)

The continuity between intracardiac mesenchyme and coronary artery SMCs through a pericyte-like intermediate state is consistent with previous developmental studies showing that proximal coronary artery SMCs originate from preotic NCCs and may arise through pericyte intermediates[8,21]*.*

(P18, L24-27)

- The authors should provide more detail on how they identified bifurcation points and more clearly explain the transition from intracardiac mesenchyme to smooth muscle cells (SMC). Additionally, they should clarify what distinguishes the three clusters (C4, C23, C27) in terms of transcription programs, marker expression, or functional states, to better support their proposed differentiation trajectories.

To clarify the distinctions among the three SMC clusters (C4, C23, C27), we added a heatmap showing differentially expressed genes, violin plots for the mature SMC marker *Myh11*, and feature plots with immunostaining images for *Myh11*, *Sost*, and *Reln* expression (Figure S4). These additional analyses further validate the molecular and spatial characteristics of the three SMC clusters. Based on these data, we revised the relevant section of the Results as follows:

The SMC clusters, which were continuous with the pharyngeal mesenchyme via transitional populations in the UMAP, were identified by high expression of the mature SMC marker Myh11 (Figure 3i). Differential gene expression analysis further distinguished individual clusters (Figure S4a-g). Among these, C27 displayed a transcriptomic profile characteristic of the great artery SMCs, including high expression of Sost (Figure S3j). C4 was enriched for Tfap2b and Ptger4 (Figure S3j), markers of the ductus arteriosus SMCs[21,22]*, supporting its annotation. C0 and C7 likely represent transitional states between pharyngeal mesenchyme and differentiated lineages, potentially bifurcating toward great artery SMCs or cardiac cushion mesenchyme (Figure S3a*

and Table S6). C23 was characterized by high expression of Gja4, a marker of coronary artery SMCs, along with pericyte markers Kcnj8 and Rgs5 (Figure 3j and Figure S3k), corresponding to the cluster similarly annotated by Chen et al[14]*. In addition, C23 was also distinguished from C4 and C27 by its expression of Reln (Figure S4d).*

Immunostaining supported these cluster annotations. Sost expression is observed in great artery SMCs but not in ductus arteriosus and coronary artery SMCs, whereas Myh11 expression was higher in ductus arteriosus and coronary artery SMCs than in aortic SMCs (Figure S4f-m). Furthermore, Reln expression was restricted to coronary artery SMCs (Figure S4n-s).

(P8, L30 – P9, L14)

To further clarify the identification of bifurcation points and the transition from intracardiac mesenchyme to SMCs, we additionally stratified the integrated UMAP according to distinct *Hox* expression profiles and inferred lineage trajectories corresponding to different neural crest and pharyngeal arch origins (Figure S9). Based on the inferred differentiation trajectory from C18 to C23 (P9, L15–19), together with the identification of C2 and C10 as *Sox9*^{high} and *Scx*^{high} intracardiac cushion mesenchymal populations contributing to coronary artery SMCs (P17, L27–33), we incorporated these lineage relationships into the schematic model presented in Figure 9.

Minor comments:

- The authors convincingly demonstrate a switch in Meis-binding motifs across NCC populations, supporting a model in which cardiac cushion-associated NCCs transition from Hox-dependent to Hox-independent transcriptional regulation via alternative cofactor interactions and DNA-binding preferences. However, the authors should provide evident on whether GATA motifs are enriched within Meis peaks, as this could further clarify potential cooperative interactions during this transition.

Although GATA-binding motifs were enriched within Meis-associated open chromatin regions in intracardiac NCCs compared with many other motifs, a substantial proportion of GATA motifs were located in peaks distinct from those containing Meis motifs. This observation raises the possibility that GATA and Meis transcription factors may cooperate through interactions across separate regulatory elements to modulate enhancer activity. However, we did not directly investigate this possibility in the present study. Instead, we found that the Meis-associated peaks

identified in intracardiac NCCs, including the distal *Sox9* enhancer containing a Meis2-binding site (chr11:112850240–112851186), more prominently contained several other consensus motifs, including predicted Hand2- and Nfatc1-binding sites. To our knowledge, however, these transcription factors have not previously been described as non-Hox cofactors of Meis proteins. Their potential recruitment to this enhancer, as well as possible cooperative interactions with Meis transcription factors during intracardiac NCC differentiation, was not examined in the current study and remains an important subject for future investigation.

- In Figure 5 panels g, j and k are difficult to interpret. The authors should provide clearer annotations, labeling, or additional explanations to improve readability and facilitate understanding of the data.

We added the annotations to UMAP in Figure 6h (and 6i) corresponding to Figure 4a and included color bars in Figure 6k as well as in 6i. To further improve readability and facilitate understanding of the data, we added the explanation of the perturbation scores in the legends for Figure 6i and k.

(original)

(g) Pseudotime trajectory analysis of integrated NCC clusters inferred using CellOracle.

(h-k) Sox9 (h, i) and Osr1 (j, k) knockout simulation presented as altered differentiation vector flows (h, j) and perturbation scores which was inner product of perturb simulation (i, k).

(revised)

(g) Pseudotime developmental flow of integrated NCC clusters from the neural tube, inferred using CellOracle and projected onto the UMAP space shown in Figure 4a.

(h-k) Sox9 (h, i) and Osr1 (j, k) knockout simulations presented as altered differentiation vector flows (h, j) and perturbation scores, defined as the inner product between the simulated perturbation vectors and the original developmental flow (i, k). Green and magenta color bars indicate normal developmental flow and reverse flow induced by perturbation of the indicated genes, respectively.

(P41, L3–L9)

- In Figure 6, results support the role of hexameric Meis-binding motif-containing region as a

distal enhancer of Sox9. The authors should provide additional results from a ChIP-qPCR experiment to further validate this model.

We attempted ChIP-seq experiments on O7-1 neural crest cell line using two different anti-Meis antibodies. However, we were unable to detect specific binding of Meis proteins to this enhancer region, although the luciferase assays clearly demonstrated the enhancer activity that was significantly attenuated by deletion of the Meis-binding motif. This discrepancy may reflect differences between endogenous chromatin and plasmid-based reporter contexts, including epigenetic modifications and chromatin accessibility. We are now investigating experimental conditions that would allow direct verification of endogenous Meis binding to this region.

- Panel l in Figure S3 requires better annotation.

We added annotations including the aorta, pulmonary valve, left coronary artery, and its septal branch.

- Correct the typo errors in Figure 5a.

The typographical errors “consercvation” and “Visuzalization” were corrected to “conservation” and “Visualization”, respectively.

- The authors should refer to previous studies showing the role of Hoxa1 and Hoxb1 in the development of great arteries or semilunar valves.

We have added the following discussion on the roles of anterior *Hox* genes in cardiovascular development, together with appropriate references:

The regional identities of pharyngeal NCCs that contribute to cardiac development are established by Hox genes and their associated regulatory networks. Genetic studies have demonstrated essential roles for the anterior Hox genes in patterning the pharyngeal arch artery system and semilunar valve structures. Loss of Hoxa1 and Hoxb1 results in severe defects in pharyngeal arch artery development[42], whereas ectopic or sustained expression of Hoxb1 in NCCs disrupts cardiovascular morphogenesis and causes malformations of the great arteries and

semilunar valves[43]. Likewise, Hoxa3 contributes to proper patterning of the pharyngeal arch region and its NCC-derived derivatives[44,45]. These findings underscore the importance of precise spatial and temporal regulation of Hox genes during cardiovascular development. Among pharyngeal NCCs contributing to cardiac development, cushion-independent NCC derivatives (great artery SMCs and the aorticopulmonary septum) retain their origin-specific Hox-codes. In contrast, cushion-associated NCC derivatives (coronary artery SMCs and valvular/subvalvular interstitial cells) downregulate Hox expression and transition toward region-specific GRNs involving TFs such as Tbx20 and Gata4, whose expression is known to be induced by BMP signaling in cardiomyocytes[46,47]. Bmp2 and Bmp4 are expressed in the regions of the pericardial reflection traversed by NCCs en route to the cardiac cushion[48]. Together, these observations suggest that appropriate repression of Hox programs, coupled with activation of cardiac-specific regulatory networks, is required for normal differentiation of cushion-associated NCC derivatives.
(P18, L30 – P19, L16)

42. *Roux, M. et al. Hoxa1 and Hoxb1 are required for pharyngeal arch artery development. Mech. Dev. 143, 1–8 (2017).*
43. *Zaffran, S., Odelin, G., Stefanovic, S., Lescroart, F. & Etchevers, H. C. Ectopic expression of Hoxb1 induces cardiac and craniofacial malformations. genesis 56, (2018).*
44. *Chisaka, O. & Capecchi, M. R. Regionally restricted developmental defects resulting from targeted disruption of the mouse homeobox gene hox-1.5. Nature 350, 473–479 (1991).*
45. *Kameda, Y., Watari-Goshima, N., Nishimaki, T. & Chisaka, O. Disruption of the Hoxa3 homeobox gene results in anomalies of the carotid artery system and the arterial baroreceptors. Cell Tissue Res. 311, 343–352 (2003).*

Referees cross-commenting

Having read the comments of the other reviewers, I totally agree with them. All our comments converge and should allow the authors to improve their manuscript.

Reviewer #3 (Significance (Required)):

The study provides high-resolution spatial and temporal mapping of NCC-derived populations and proposes mechanistic insights into Hox-dependent versus Hox-independent transcriptional regulation, as well as a Meis2-Sox9-Scx gene regulatory network contributing to smooth muscle and semilunar valve formation.

Strengths and limitations: The datasets are rich and well-integrated, offering valuable resources for the field. However, several key conclusions rely on correlative analyses and heterogeneous datasets, making some claims speculative. Technical details, such as FACS gating, low representation of cardiomyocytes, and interpretation of RNA velocity, require further clarification, which currently limits the strength of the mechanistic inferences.

Advance: This work advances the understanding of NCC lineage diversification and gene regulatory dynamics in cardiopharyngeal development, particularly highlighting potential transcriptional switches and intermediate progenitor states that guide structural formation in the heart.

Audience: The study will be of interest to researchers in developmental biology, cardiovascular biology, and single-cell multi-omics, particularly those studying neural crest cell differentiation and cardiac morphogenesis.

Again, thank you for giving us the opportunity to strengthen our manuscript with your valuable comments and queries. We have worked hard to incorporate your feedback and hope that these revisions persuade and satisfy you.

Dear Dr. Kurihara,

Thank you for submitting a revised version of your Review Commons manuscript to The EMBO journal. Your study has now been seen by two of the original referees, who find that their previous concerns have been sufficiently addressed and now recommend publication of the manuscript after minor textual revisions.

In addition, there remain a few editorial and formatting points that need to be addressed before I can extend the official acceptance of the manuscript:

1. Please note that it is The EMBO Journal policy for the transcript of the editorial process (containing referee reports and your response letters) to be published as an online supplement to each paper. If you should prefer removal of any referee-only figures included in the point-by-point response(s), e.g. because they may still be used for future publication or because they have been reproduced from published work by others, please do let us know immediately via response email.
More information is available here: https://www.embopress.org/transparent-process#Review_Process
2. Please submit a complete author checklist, which you can download from our author guidelines (https://media.springernature.com/original/springer-cms/rest/v1/content/27825796/data/v1). Please insert information in the checklist that is also reflected in the manuscript. The completed author checklist will also be part of the Review Process File.
3. Please upload the main and EV figures as individual production quality figure files in the .eps, .tif,or .jpg format (one file per figure). Please remove the figure from the manuscript text file.
4. Please update the order and headings of the sections in the manuscript text to: Abstract / Introduction / Results / Discussion / Methods / Data Availability / Acknowledgements / Disclosure and Competing Interests Statement / References / Figure Legends / Expanded View Figure Legends.
5. Please remove the "Materials and Correspondence" section.
6. Please ensure that the list of funders entered into our system is complete and accurate; the list will be linked to the corresponding database in PubMed. Currently, JPMJCR13W2, 24K11186, 16H06279, JP22H0492, Mitsubishi Foundation, the Fugaku Foundation and RIKAKEN HD Life science research grant are not included in our online system, please add if relevant.
7. CRediT has replaced the traditional author contributions section because it offers a systematic, machine-readable author contributions format that allows for more effective research assessment. Please remove the Authors Contributions from the manuscript and use the free text boxes beneath each contributing author's name in our online submission system to add specific details on the author's contribution. More information is available in our guide to authors.
8. Please update references according to The EMBO Journal style - it should be alphabetical; where there are more than 10 authors on a paper, the first 10 should be listed, followed by 'et al.'
9. In the manuscript text, a reference should be cited by author and year of publication; no more than two authors may be cited per reference; 'et al' should be used if there are more than two authors (i.e. Smith & Jones, 2003; Smith et al, 2000).
10. Please merge the "Data Availability" section with "Code Availability". Please include resolvable URLs for datasets (DRA015815, DRA015814, DRA014005, DRA012897, DRA010734, GEAD-430, GEAD-686, A-GEAD-2, GSE201417) in the "Data Availability" section.
11. Figure panels Fig 1M, Fig 4F,G,H are not mentioned in the manuscript text, please check and correct.
12. Please rename Suppl. Figures and Suppl. Tables, the correct nomenclature is Figure EV1 - EV13 and Dataset EV1 - Dataset EV10.
13. Please add Dataset legends to the corresponding files in another tab/sheet.
14. All Materials and Methods need to be described in the main text using our 'Structured Methods' format.
According to this format, the Methods section includes a Reagents and Tools Table (listing key reagents, experimental models, software and relevant equipment and including their sources and relevant identifiers) followed by a Methods and Protocols section describing the methods, ideally using a step-by-step protocol format. The aim is to facilitate adoption of the methodologies across labs.
Please download and fill our Reagents and Tools Table template (.docx), which you can find in our author guidelines: https://media.springernature.com/original/springer-cms/rest/v1/content/27825802/data/v1
When submitting your revised manuscript, please do not include the Reagents and Tools Table in the Methods section of the manuscript, but upload it as a separate file choosing the file type "Reagent Table".
An example of a Method paper with Structured Methods can be found here: https://link.springer.com/article/10.15252/msb.20178071.
15. At EMBO Press we ask authors to provide source data for the main manuscript figures. You will receive a separate email with instructions for providing source data with your revised manuscript, including how to upload and organise the files. Please submit these files with the final version.
16. Our data editors have flagged the following issues in figure legends that need correcting:
- Please indicate the statistical test used for data analysis in the legends of figure 7C.
- Please define the box plots in terms of centre, minima, maxima, and whiskers, bounds of box and percentile in the legends of figure 7C.
- Please provide information on the number and nature of replicates in the legends of figures 6B,C,E,F; 7C.
- Please note define the scale bar for figures 1A.
17. Papers published in The EMBO Journal are accompanied online by a 'Synopsis' to enhance discoverability of the

manuscript. It consists of A) a short (1-2 sentences) summary of the findings and their significance, B) 3-4 bullet points highlighting key results (the highlights currently included in the manuscript can be used here) and C) a synopsis image that is 550x300 pixels large (width x height, jpeg or png format). You can either show a model or key data in the synopsis image. Please note that the image size is rather small and that text needs to be readable at the final size.

Please let me know if you have any questions regarding any of these points. You can use the link below to upload the revised files.

Thank you again for giving us the chance to consider your manuscript for The EMBO Journal. I look forward to receiving the final version.

Yours sincerely,

Ieva Gailite

Ieva Gailite, PhD
Senior Scientific Editor
The EMBO Journal
Meyerhofstrasse 1
69117 Heidelberg
i.gailite@embojournal.org

Instructions for preparing your revised manuscript:

Read our guidance for manuscript revisions and related editorial policies: https://link.springer.com/journal/44318/submission-guidelines#cms-Revised-submissions

Please remember: Digital image enhancement is acceptable practice, as long as it accurately represents the original data and conforms to community standards. If a figure has been subjected to significant electronic manipulation, this must be noted in the figure legend or in the 'Materials and Methods' section. The editors reserve the right to request original versions of figures and the original images that were used to assemble the figure.

Revision to The EMBO Journal should be submitted online within 90 days, unless an extension has been requested and approved by the editor; please click on the link below to submit the revision online before 5th Oct 2026:

Link Not Available

Referee #1:

The authors have extensively revised and reorganised their manuscript, inlcuding new analysis. This important and data rich study significantly extends understanding of how neural crest cells contribute to cardiovascular structures, in addition providing a valuable ressource to the field through an accessible browser.

I have one remaining point - concerning the putative acquisiton of cardiomyocyte fate, the authors should add to the text on page 6 that this is observed in only 6 genetically traced NCC cells (approximately 0.1%), according to their reply to the original comment.

Referee #2:

The authors have satisfactorily responded to my comments. They have addressed my concerns by improving the annotations, clarifying the text, results and discussion, including the limitations of their computational approaches in inferring true lineage relationships. Overall, this work advances our understanding of NCC diversification and cardiac development.

Referee #3:

In this revised version, the authors have conscientiously addressed my comments. They have modified the manuscript and expanded the discussion of several points, resulting in a stronger and more convincing support of their conclusions. For instance, the revised Figure 1 and Methods section now provide details on the FACS gating strategy and sorting conditions. The authors also better explain how the cardiopharyngeal tissue and OFT region were dissected. Refinement of the clustering analysis has improved the resolution and interpretability of the identified populations, and the mesenchymal clusters are now better annotated. As suggested in my previous comments, separate UMAPs displaying NCC and non-NCC populations is now shown in Figure 1. The authors also applied an upper cutoff to improve the spatial visualization of putative EYFP-positive cells in the revised Figure 2. In addition, a sentence has been included to clarify the issue of the detection of NCC within cardiomyocyte clusters (p6). The limitations of RNA-velocity analysis are now better explained, and Figure 3 has been revised to avoid confusion regarding the continuity between C18 and C23. They also provide more details on how they distinguished the 3 SMC clusters (Fig S4). Finally, the authors addressed my minor comments by revising Figure 5 and adding text to the discussion section.
Overall, I find that the revised manuscript has been substantially improved, and the data now convincing support the conclusion proposed by the authors.

***

Rev_Com_number: RC-2025-03308
New_manu_number: EMBOJ-2026-124818-T
Corr_author: Kurihara
Title: Hox-Meis-relayed gene regulatory transition underlies cardiopharyngeal neural crest diversification

Responses to the reviewers

thank all of the reviewers for their thoughtful and constructive comments throughout the review process. We are delighted that the revised manuscript has addressed your concerns and met with your approval. Our responses to the remaining comments are provided below in **blue**.

To Referee #1:

The authors have extensively revised and reorganised their manuscript, including new analysis. This important and data rich study significantly extends understanding of how neural crest cells contribute to cardiovascular structures, in addition providing a valuable resource to the field through an accessible browser.

I have one remaining point - concerning the putative acquisition of cardiomyocyte fate, the authors should add to the text on page 6 that this is observed in only 6 genetically traced NCC cells (approximately 0.1%), according to their reply to the original comment.

We thank the reviewer for this helpful suggestion. We have revised the corresponding sentence as follows:

Only six genetically traced NCCs (approximately 0.1% of all NCCs) were detected within cardiomyocyte clusters, which were predominantly composed of non-NCCs, …
(P6, L19-21)

Finally, we sincerely thank the reviewer for the careful and thorough evaluation of our work and for the many constructive comments provided throughout the review process. Your insightful suggestions have substantially improved the clarity and quality of our manuscript.

To Referee #2:

The authors have satisfactorily responded to my comments. They have addressed my concerns by improving the annotations, clarifying the text, results and discussion, including the limitations of

their computational approaches in inferring true lineage relationships. Overall, this work advances our understanding of NCC diversification and cardiac development.

We sincerely thank the reviewer for the careful and thorough evaluation of our work and for the many constructive comments provided throughout the review process. Your insightful suggestions have substantially improved the clarity and quality of our manuscript.

To Referee #3:

In this revised version, the authors have conscientiously addressed my comments. They have modified the manuscript and expanded the discussion of several points, resulting in a stronger and more convincing support of their conclusions. For instance, the revised Figure 1 and Methods section now provide details on the FACS gating strategy and sorting conditions. The authors also better explain how the cardiopharyngeal tissue and OFT region were dissected. Refinement of the clustering analysis has improved the resolution and interpretability of the identified populations, and the mesenchymal clusters are now better annotated. As suggested in my previous comments, separate UMAPs displaying NCC and non-NCC populations is now shown in Figure 1. The authors also applied an upper cutoff to improve the spatial visualization of putative EYFP-positive cells in the revised Figure 2. In addition, a sentence has been included to clarify the issue of the detection of NCC within cardiomyocyte clusters (p6). The limitations of RNA-velocity analysis are now better explained, and Figure 3 has been revised to avoid confusion regarding the continuity between C18 and C23. They also provide more details on how they distinguished the 3 SMC clusters (Fig S4). Finally, the authors addressed my minor comments by revising Figure 5 and adding text to the discussion section.
Overall, I find that the revised manuscript has been substantially improved, and the data now convincing support the conclusion proposed by the authors.

We sincerely thank the reviewer for the careful and thorough evaluation of our work and for the many constructive comments provided throughout the review process. Your insightful suggestions have substantially improved the clarity and quality of our manuscript.

1st Revision - Editorial Decision 20th Jul 2026

Dear Dr. Kurihara,

Thank you for incorporating the final editorial and formatting requests in the manuscript. I am now pleased to inform you that your manuscript has been accepted for publication. Congratulations with a nice study!

Before we forward your manuscript to our publishers, there are a couple of aspects that I still need your input on:

1. We noted that resolvable URLs to the individual datasets have not been added to the Data Availability section. Please send me the corresponding links and we will incorporate them in the manuscript for you. Please also note that these datasets will need to be publicly accessible latest upon online publication of the study, but optimally already at the proofs stage.

2. We would like to propose some edits in the manuscript title, abstract and synopsis. We have also selected a short blurb that will accompany the title of your manuscript in our online table of contents. Please take a look at the proposed textual changes in the attached text file - any corrections or edits from your side are very welcome.

Your manuscript will be processed for publication by EMBO Press. It will be copy edited and you will receive page proofs prior to publication. Please note that you will be contacted by Springer Nature Author Services to complete licensing and payment information.

You may qualify for financial assistance for your publication charges - either via a Springer Nature fully open access agreement or an EMBO initiative. Check your eligibility: https://link.springer.com/journal/44318/how-to-publish-with-us

Should you be planning a Press Release on your article, please notify the production team via this form in order to coordinate publication and release dates: https://forms.cloud.microsoft/e/eH4A8JVP6g

If you have any questions, please do not hesitate to contact the Editorial Office. I look forward to hearing from you soon on the final textual changes.
Thank you for this interesting contribution to The EMBO Journal!

Best wishes,

Ieva

Ieva Gailite, PhD
Senior Scientific Editor
The EMBO Journal
Meyerhofstrasse 1
69117 Heidelberg
i.gailite@embojournal.org

***
Rev_Com_number: RC-2025-03308
New_manu_number: EMBOJ-2026-124818R
Corr_author: Kurihara
Title: Hox-Meis-relayed gene regulatory transition underlies cardiopharyngeal neural crest diversification

EMBO Press Author Checklist

Corresponding Author Name: Akiyasu Iwase, Hiroki Kurihara	
Journal Submitted to: The EMBO Journal	
Manuscript Number: EMBOJ-2026-124818-T	

USEFUL LINKS FOR COMPLETING THIS FORM
The EMBO Journal - Author Guidelines
EMBO Reports - Author Guidelines
Molecular Systems Biology - Author Guidelines
EMBO Molecular Medicine - Author Guidelines

Reporting Checklist for Life Science Articles (updated January
This checklist is adapted from Materials Design Analysis Reporting (MDAR) Checklist for Authors. MDAR establishes a minimum set of requirements in transparent reporting in the life sciences (see Statement of Task: 10.31222/osf.io/9sm4x). Please follow the journal's guidelines in preparing your
Please note that a copy of this checklist will be published alongside your article.

Abridged guidelines for figures
1. Data
The data shown in figures should satisfy the following conditions:
- the data were obtained and processed according to the field's best practice and are presented to reflect the results of the experiments in an accurate and unbiased manner.
- ideally, figure panels should include only measurements that are directly comparable to each other and obtained with the same assay.
- plots include clearly labeled error bars for independent experiments and sample sizes. Unless justified, error bars should not be shown for technical
- if n<5, the individual data points from each experiment should be plotted. Any statistical test employed should be justified.
- Source Data should be included to report the data underlying figures according to the guidelines set out in the authorship guidelines on Data

2. Captions
Each figure caption should contain the following information, for each panel where they are relevant:
- a specification of the experimental system investigated (eg cell line, species name).
- the assay(s) and method(s) used to carry out the reported observations and measurements.
- an explicit mention of the biological and chemical entity(ies) that are being measured.
- an explicit mention of the biological and chemical entity(ies) that are altered/varied/perturbed in a controlled manner.
- the exact sample size (n) for each experimental group/condition, given as a number, not a range;
- a description of the sample collection allowing the reader to understand whether the samples represent technical or biological replicates (including how many animals, litters, cultures, etc.).
- a statement of how many times the experiment shown was independently replicated in the laboratory.
- definitions of statistical methods and measures:
  - common tests, such as t-test (please specify whether paired vs. unpaired), simple $\chi 2$ tests, Wilcoxon and Mann-Whitney tests, can be unambiguously identified by name only, but more complex techniques should be described in the methods section;
  - are tests one-sided or two-sided?
  - are there adjustments for multiple comparisons?
  - exact statistical test results, e.g., P values = x but not P values < x;
  - definition of 'center values' as median or average;
  - definition of error bars as s.d. or s.e.m.

Please complete ALL of the questions below.
Select "Not Applicable" only when the requested information is not relevant for your study.

Materials

Newly Created Materials	Information included in the manuscript?	In which section is the information available? (Reagents and Tools Table, Materials and Methods, Figures, Data Availability Section)
New materials and reagents need to be available; do any restrictions apply?	Yes	Materials and Methods, Reagent and Tools table

Antibodies	Information included in the manuscript?	In which section is the information available? (Reagents and Tools Table, Materials and Methods, Figures, Data Availability Section)
For **antibodies** provide the following information:<br>- Commercial antibodies: RRID (if possible) or supplier name, catalogue number and or/clone number<br>- Non-commercial: RRID or citation	Yes	Materials and Methods, Reagent and Tools table

DNA and RNA sequences	Information included in the manuscript?	In which section is the information available? (Reagents and Tools Table, Materials and Methods, Figures, Data Availability Section)
**Short novel DNA or RNA including primers, probes**: provide the sequences.	Yes	Reagent and Tools table

Cell materials	Information included in the manuscript?	In which section is the information available? (Reagents and Tools Table, Materials and Methods, Figures, Data Availability Section)
**Cell lines:** Provide species information, strain. Provide accession number in repository **OR** supplier name, catalog number, clone number, and/**OR** RRID.	Yes	Materials and Methods, Reagent and Tools table
**Primary cultures:** Provide species, strain, sex of origin, genetic modification status.	Not Applicable	
Report if the cell lines were recently **authenticated** (e.g., by STR profiling) and tested for mycoplasma contamination.	Not Applicable	

Experimental animals	Information included in the manuscript?	In which section is the information available? (Reagents and Tools Table, Materials and Methods, Figures, Data Availability Section)
**Laboratory animals or Model organisms:** Provide species, strain, sex, age, genetic modification status. Provide accession number in repository **OR** supplier name, catalog number, clone number, **OR** RRID.	Yes	Materials and Methods, Reagent and Tools table
**Animal observed in or captured from the field:** Provide species, sex, and age where possible.	Not Applicable	
Please detail **housing and husbandry conditions**.	Yes	Materials and Methods

Plants and microbes	Information included in the manuscript?	In which section is the information available? (Reagents and Tools Table, Materials and Methods, Figures, Data Availability Section)
**Plants:** provide species and strain, ecotype and cultivar where relevant, unique accession number if available, and source (including location for collected wild specimens).	Not Applicable	
**Microbes:** provide species and strain, unique accession number if available, and source.	Not Applicable	

Human research participants	Information included in the manuscript?	In which section is the information available? (Reagents and Tools Table, Materials and Methods, Figures, Data Availability Section)
If collected and within the bounds of privacy constraints report on age, sex and gender or ethnicity for all study participants.	Not Applicable	

Core facilities	Information included in the manuscript?	In which section is the information available? (Reagents and Tools Table, Materials and Methods, Figures, Data Availability Section)
If your work benefited from core facilities, was their service mentioned in the acknowledgments section?	Yes	Materials and Methods

Design

Study protocol	Information included in the manuscript?	In which section is the information available? (Reagents and Tools Table, Materials and Methods, Figures, Data Availability Section)
If study protocol has been **pre-registered, provide DOI in the manuscript**. For clinical trials, provide the trial registration number **OR** cite DOI.	Not Applicable	
Report the **clinical trial registration number** (at ClinicalTrials.gov or equivalent), where applicable.	Not Applicable	

Laboratory protocol	Information included in the manuscript?	In which section is the information available? (Reagents and Tools Table, Materials and Methods, Figures, Data Availability Section)
Provide DOI OR other citation details if **external detailed step-by-step protocols** are available.	Not Applicable	

Experimental study design and statistics	Information included in the manuscript?	In which section is the information available? (Reagents and Tools Table, Materials and Methods, Figures, Data Availability Section)
Include a statement about **sample size** estimate even if no statistical methods were used.	Yes	Figure legends
Were any steps taken to minimize the effects of subjective bias when allocating animals/samples to treatment (e.g. **randomization procedure**)? If yes, have they been described?	Not Applicable	
Include a statement about **blinding** even if no blinding was done.	Not Applicable	
Describe **inclusion/exclusion criteria** if samples or animals were excluded from the analysis. Were the criteria pre-established?<br><br>If sample or data points were omitted from analysis, report if this was due to attrition or intentional exclusion and provide justification.	Yes	Materials and Methods
For every figure, are **statistical tests** justified as appropriate? Do the data meet the assumptions of the tests (e.g., normal distribution)? Describe any methods used to assess it. Is there an estimate of variation within each group of data? Is the variance similar between the groups that are being statistically compared?	Yes	Figure legends

Sample definition and in-laboratory replication	Information included in the manuscript?	In which section is the information available? (Reagents and Tools Table, Materials and Methods, Figures, Data Availability Section)
In the figure legends: state number of times the experiment was **replicated** in laboratory.	Yes	Figure legends
In the figure legends: define whether data describe **technical or biological replicates**.	Yes	Figure legends

Ethics

Ethics	Information included in the manuscript?	In which section is the information available? (Reagents and Tools Table, Materials and Methods, Figures, Data Availability Section)
Studies involving **human participants**: State details of **authority granting ethics approval** (IRB or equivalent committee(s), provide reference number for approval.	Not Applicable	
Studies involving **human participants**: Include a statement confirming that **informed consent** was obtained from all subjects and that the experiments conformed to the principles set out in the WMA Declaration of Helsinki and the Department of Health and Human Services Belmont Report.	Not Applicable	
Studies involving **human participants:** For publication of **patient photos**, include a statement confirming that consent to publish was obtained.	Not Applicable	
Studies involving experimental **animals**: State details of **authority granting ethics approval** (IRB or equivalent committee(s), provide reference number for approval. Include a statement of compliance with ethical regulations.	Yes	Materials and Methods
Studies involving **specimen and field samples:** State if relevant **permits** obtained, provide details of authority approving study; if none were required, explain why.	Not Applicable	

Dual Use Research of Concern (DURC)	Information included in the manuscript?	In which section is the information available? (Reagents and Tools Table, Materials and Methods, Figures, Data Availability Section)
Could your study fall under dual use research restrictions? Please check biosecurity documents and list of **select agents and toxins** (CDC): https://www.selectagents.gov/sat/list.htm	Not Applicable	
If you used a select agent, is the security level of the lab appropriate and reported in the manuscript?	Not Applicable	
If a study is subject to dual use research of concern regulations, is the name of the **authority granting approval and reference number** for the regulatory approval provided in the manuscript?	Not Applicable	

Reporting

The MDAR framework recommends adoption of discipline-specific guidelines, established and endorsed through community initiatives. Journals have their own policy about requiring specific guidelines and recommendations to complement MDAR.

Adherence to community standards	Information included in the manuscript?	In which section is the information available? (Reagents and Tools Table, Materials and Methods, Figures, Data Availability Section)
State if relevant guidelines or checklists (e.g., **ICMJE, MIBBI, ARRIVE, PRISMA**) have been followed or provided.	Not Applicable	
For **tumor marker prognostic studies,** we recommend that you follow the **REMARK** reporting guidelines (see link list at top right). See author guidelines, under 'Reporting Guidelines'. Please confirm you have followed these guidelines.	Not Applicable	
For **phase II and III randomized controlled trials**, please refer to the **CONSORT** flow diagram (see link list at top right) and submit the CONSORT checklist (see link list at top right) with your submission. See author guidelines, under 'Reporting Guidelines'. Please confirm you have submitted this list.	Not Applicable	

Data Availability

Data availability	Information included in the manuscript?	In which section is the information available? (Reagents and Tools Table, Materials and Methods, Figures, Data Availability Section)
Have **primary datasets** been deposited according to the journal's guidelines (see 'Data Deposition' section) and the respective accession numbers provided in the Data Availability Section?	Yes	Data Availability
Were **human clinical and genomic datasets** deposited in a public access-controlled repository in accordance to ethical obligations to the patients and to the applicable consent agreement?	Not Applicable	
Are **computational models** that are central and integral to a study available without restrictions in a machine-readable form? Were the relevant accession numbers or links provided?	Yes	Data Availability
If publicly available data were reused, provide the respective **data citations in the reference list**.	Yes	Data Availability
